# Stenting techniques for coronary bifurcation disease: a systematic review and network meta-analysis demonstrates superiority of double-kissing crush in complex lesions

**DOI:** 10.1007/s00392-021-01979-9

**Published:** 2021-12-04

**Authors:** Rui Wang, Yaodong Ding, Jiaxin Yang, Kexin Wang, Wen Gao, Zhe Fang, Yujie Zhou, Hailong Ge

**Affiliations:** 1 Department of Cardiology, Beijing Anzhen Hospital, Capital Medical University, Beijing, 100029 People’s Republic of China; 2 Department of Cardiology, Bayannaoer City Hospital, Inner Mongolia, 015002 People’s Republic of China; 3 Department of Cardiology, Beijing Daxing District People’s Hospital, Capital Medical University Daxing Teaching Hospital, Capital Medical University, Beijing, 102699 People’s Republic of China

**Keywords:** Coronary bifurcation disease, Stenting techniques, Network meta-analysis

## Abstract

**Objective:**

This study was aimed to compare different stenting techniques for coronary bifurcation disease (CBD).

**Background:**

Percutaneous coronary intervention (PCI) remains controversial for CBD; over the years, several stent techniques for bifurcation lesions have been used. Current guidelines recommend a provisional single-stent strategy as the preferred method for coronary artery bifurcation lesions. However, several randomized controlled trials (RCT) indicated that two-stent techniques showed better clinical outcomes.

**Methods:**

We systematically searched Embase, PubMed, and Web of Science to include RCTs. The primary endpoint was the major adverse cardiovascular event (MACE). Secondary outcomes were cardiac death, myocardial infarction (MI), target-lesion or target-vessel revascularization (TLR or TVR), and definite or probable stent thrombosis (ST). Finally, we used 26 RCTs and a total of 7257 individuals were randomly assigned to one of the 6 stent techniques and included in this network meta-analysis.

**Results:**

In our network meta-analysis, double-kissing (DK) crush was significantly more superior to other 5 stent techniques in MACEs: OR vs. provisional 0.40 (95% CI 0.28–0.55); vs. culotte 0.40 (95% CI 0.26–0.60). DK crush ranked the most effective treatment for MACE (100%), MI (75%), ST (83%), and TLR (100%) in the rank probabilities analysis. In patients with complex bifurcation lesion defined by DEFINITION criteria, DK crush was notably more efficacious than provisional, culotte, and T-stenting/T-stenting and protrusion (TAP) in MACEs (OR vs. provisional 0.26, 95% CI 0.13–0.52) and TLR (OR vs. provisional 0.24, 95% CI 0.10–0.58).

**Conclusion:**

Compared with other stenting techniques, DK crush had a lower incidence of MACEs in CBD. DK crush was significantly associated with a lower rate of MACEs in patients with complex bifurcation lesions defined by the DEFINITION criterion.

**Graphical abstract:**

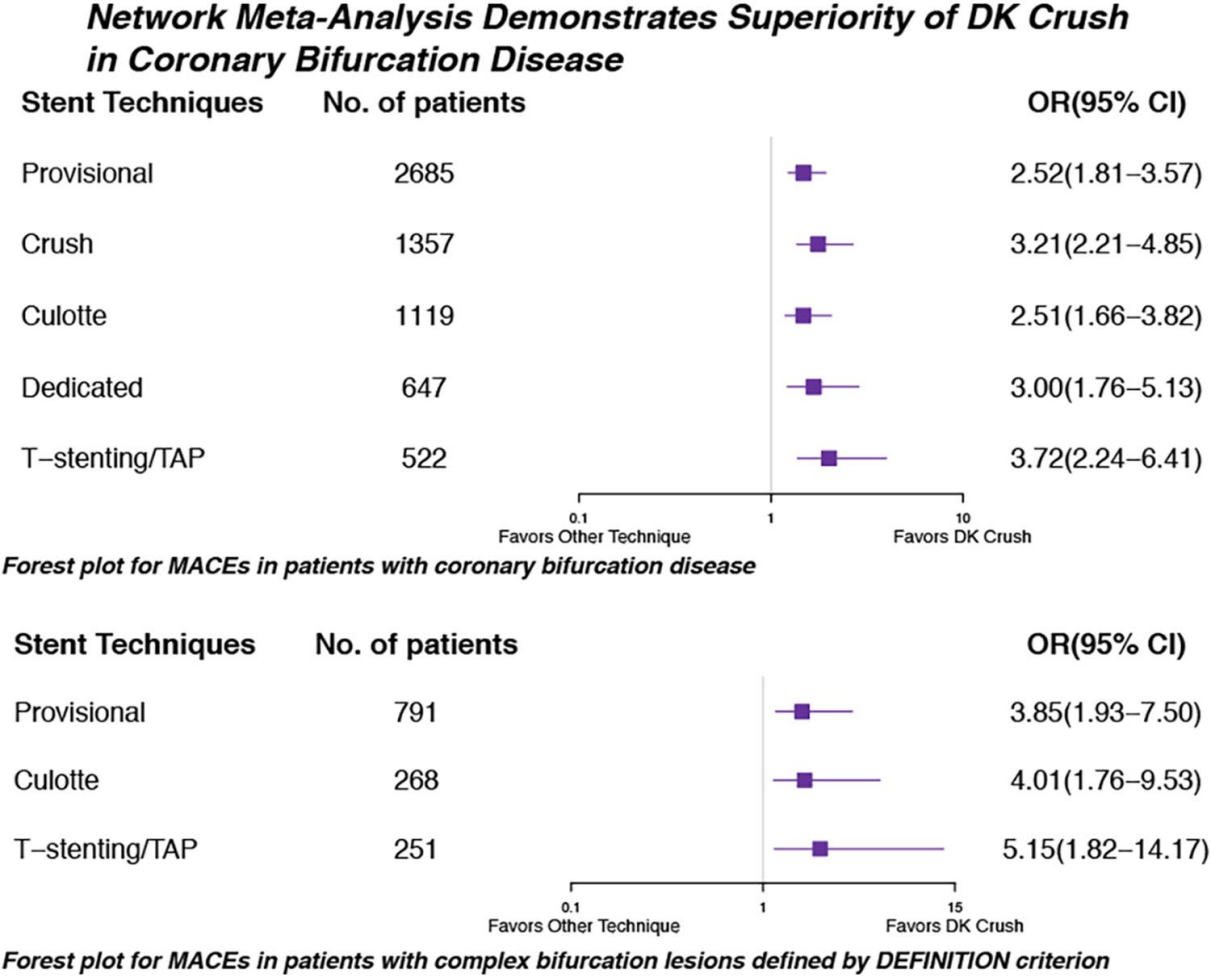

**Supplementary Information:**

The online version contains supplementary material available at 10.1007/s00392-021-01979-9.

## Introduction

Bifurcation lesions is defined as coronary artery stenosis that occurs adjacent to and/or involving in the origin of an important side branch (SB) that the operator does not want to lose. All currently adopted definitions are based on the involvement of SB in the main branch (MB) lesion, and usually take into consideration the diameter of the SB [1]. The MEDINA classification for bifurcation lesion was considered to be the simplest to understand and use, and was available to everybody [2]. It uses binary descriptors to indicate whether there is any lesion in each branch of the bifurcation. However, it could not provide sufficient information about the true complexity of a given bifurcation lesion.

Percutaneous coronary intervention (PCI) remains controversial for coronary bifurcation disease (CBD); over the years, several stent techniques for bifurcation lesions have been used. Furthermore, CBD is common, comprising 15–20% of PCIs. Current guidelines recommend a provisional single-stent strategy as the preferred method for coronary artery bifurcation lesions [3]. Nevertheless, several randomized controlled trials (RCT) indicate that two-stent techniques yield better clinical outcomes [4, 5].

We report an overview of all RCTs that compared 6 stenting techniques in terms of efficacy and clinical outcomes in the PCI for bifurcation lesions. The present study aimed to provide a clinically useful summary of the results of the network meta-analysis that can be used to guide treatment decisions during coronary bifurcation interventions.

## Methods

### Study design and selection

For our analysis, we included only RCTs that compared at least 2 different techniques of following 6 stent strategies [provisional T-stenting or single stent, T-stenting or T-stenting and protrusion (TAP), crushing, double-kissing (DK) crush, dedicated bifurcation stents, and culotte] as monotherapy in the intervention treatment of adults with CBD. We excluded RCTs that did not include any of the above techniques and the bifurcation of a chronic total occlusion (CTO).

To identify the relevant studies, we systematically searched Embase, PubMed, and Web of Science up to Apr 24, 2021. Research strategies and keywords are outlined in Supplemental Table S1. One researcher (J.Y.) checked all the titles and abstracts, and classified them whether to be included based on the criteria aforementioned. Two clinical investigators (Z.F. and W.G.) independently reviewed full text to examine eligibility criteria. The final decision to include the article was made by consensus of the team. Two independent members (R.W. and D.D.) of the reviewing team reviewed the retrieved references and abstracts, assessed the completeness of the data abstraction, and confirmed the quality rating. An experienced interventional cardiologist (H.G.) retrieved the quantitative information for outcomes and patient characteristics from selected articles.

Data were extracted to report this meta-analysis in agreement with Preferred Reporting Items for Systematic Reviews and Meta-Analyses (PRISMA) guidelines in Supplemental Table S2. Bias assessment was performed using the Cochrane Collaboration's tool in Supplemental Table S3. Extracted data included study endpoint and follow-up definition, and pre- and post-PCI lesion characteristics in supplemental materials. Ethical approval was obtained in the context of each study. This network meta-analysis was registered in PROSPERO (CRD42021250754).

### Outcome measures

The primary endpoint was the major adverse cardiovascular event (MACE), defined by each study, as the combination of all-cause death or cardiac death, target-vessel myocardial infarction (MI), stent thrombosis (ST), target lesion, or target-vessel revascularization (TLR or TVR) and coronary artery bypass graft (CABG). The primary endpoint was calculated as the sum of individual components of every single study. Endpoint and follow-up definitions of each study are presented in Supplemental Table S4.

Secondary outcomes were cardiac death, MI, TLR, or TVR and definite or probable ST. We defined stent thrombosis according to the Academic Research Consortium (ARC) criteria.

### Statistical analysis

A network meta-analysis to compare MACEs between different bifurcation PCI treatments was plotted in a network map. First, a pair-wise meta-analysis for comparing the same interventions with a random-effects model reported the effect size outcome using adjusted odds ratios (OR), with a corresponding 95% confidence interval (CI). We drew Forest plots for each pair-wise comparison. We did the analyses using Stata software v14 (StataCorp, College Station, TX).

Afterward, we did a random-effect model to compare 6 stenting techniques for CBD within a Bayesian framework with Markov-chain Monte Carlo methods in GeMTC v0.14.3, using 4 chains with over-dispersed values and Gibbs sampling on the bias of 100,000 simulation iterations. A consistency test combined direct, indirect evidence through a node-splitting model and network estimates for any given pair of treatments was performed for testing whether the model was stable. A set of 20,000 tuning iterations and vague priors were used in all models. We also assessed the probability that each PCI strategy was the treatment with the most likely to onset the endpoint event, the second, the third, and so on, using the surface under the cumulative ranking curve (SUCRA). Heterogeneity was evaluated by means of the Cochrane Q test and measured with the use of the Higgins *I*^*2*^ test.

Finally, we performed sensitivity analyses according to the following variables: true bifurcation (including only true bifurcation), bias risk (including only low-risk studies, studies without high risk), and DEFINITION criteria [6] (including complex bifurcation).

## Results

### Literature search and inclusion

**Fig. 1:**
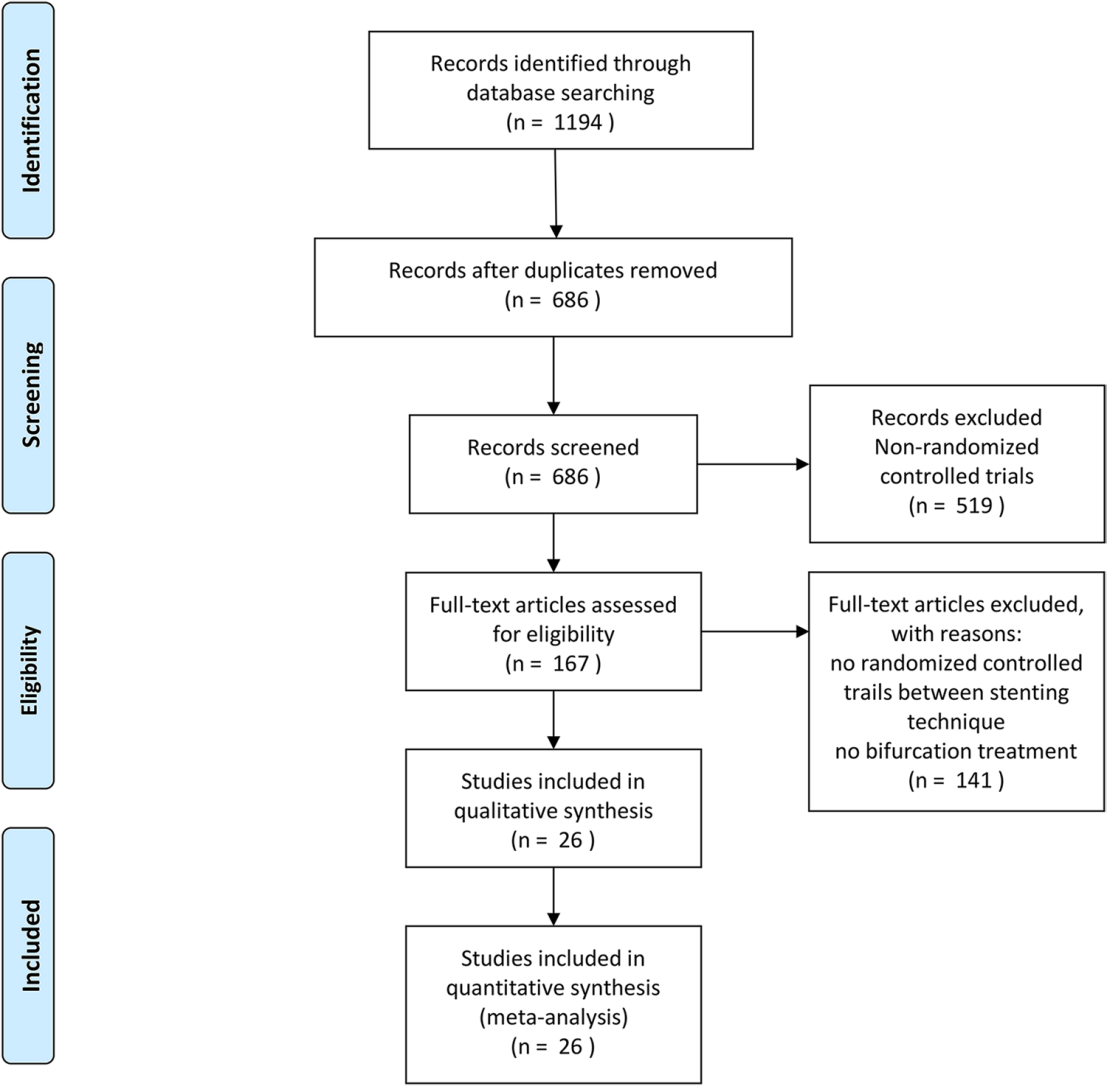
Study flow diagram

The electronic searches yielded 1194 potentially relevant studies, of which 167 potentially eligible articles were analyzed. Ultimately, we used 26 randomized controlled trials from 2004 to 2020 for the review and multiple-treatment meta-analysis. Figure 1 displays the research flow diagram and the reasons for exclusion. Overall, a total of 7257 individuals were randomly assigned to one of the 6 stent techniques and included in this network meta-analysis.

The median clinical follow-up time for 7233 patients (99.7%) was 12.0 months (range: 6 to 60 months) and 14 trials (53.8%) had a follow-up at least 12 months. The mean sample size was 139.2 patients per group (range: 22 to 349) and 16 studies (61.5%) had at least 100 patients in each group. In terms of clinical characteristics, the average age of the patients included in the analysis was 64 ± 10 years, 71% were male, 68% had hypertension, 56% had hyperlipidemia, and 22% had diabetes mellitus. In the pooled participants, 2685 patients (37%) were treated with provisional stenting, 522 patients (7%) receiving T-stenting/TAP, 1357 patients (19%) with the crush, 903 patients (12%) with DK Crush, 1119 patients (15%) patients receiving culotte, and 647 patients (9%) with dedicated bifurcation stent. The final kissing balloon (FKB) was performed in 5115 patients (70%), especially 97% in the DK Crush arm. The clinical, angiographic, and procedural characteristics are reported in Table 1. The quantitative coronary angiographic analysis of pre-and post- procedural is shown in Supplemental Table S5 and S6.

**Table 1:** Clinical, angiographic, and procedural characteristics

Study	Year	Intervention	Sample size	Age	Sex male	Hyperlipidemia	Hypertension	Diabetes mellitus	Prior MI
Colombo et al.	2004	T/TAP vs. provisional	86	63 ± 10 62 ± 9	48 (76) 21 (91)	NA	NA	13 (21) 6 (26)	NA
Pan et al.	2004	Provisional vs. T/TAP	91	61 ± 10 58 ± 11	34 (72) 38 (86)	25 (53) 18 (41)	28 (59) 25 (57)	20 (42) 17 (39)	9 (19) 17 (39)
DKCRUSH-1	2008	Crush vs DK crush	311	64 ± 9 64 ± 9	109 (70) 118 (76)	98 (63) 106 (69)	120 (77) 118 (76)	13 (8) 42 (27)	19 (12) 13 (9)
CACTUS	2009	Crush vs. provisional	350	65 ± 10 67 ± 10	142 (80) 132 (76)	113 (64) 122 (71)	125 (71) 138 (80)	42 (24) 38 (22)	79 (45) 61 (35)
THUEBIS	2009	Dedicated vs. provisional	110	67 ± 9 65 ± 11	40 (71) 42 (78)	28 (50) 35 (67)	49 (88) 42 (78)	14 (25) 14 (26)	NA
BBC ONE	2010	Provisional vs crush	500	64 ± 10 64 ± 11	192 (77) 193 (77)	188 (76) 189 (76)	142 (57) 157 (62)	31 (13) 29 (11)	57 (23) 63 (25)
Lin et al.	2010	Provisional vs. DK crush	108	61 ± 7 59 ± 7	45 (83) 41 (76)	NA	49 (91) 45 (83)	10 (19) 7 (13)	12 (22) 10 (19)
Ye et al.	2010	DK crush vs. provisional	51	64 ± 12 63 ± 10	16 (64) 19 (73)	4 (16) 3 (12)	19 (76) 19 (73)	4 (16) 5 (19)	NA
Ye et al.	2012	DK crush vs. provisional	68	64 ± 11 62 ± 10	24 (63) 23 (77)	7 (18) 6 (20)	29 (76) 20 (67)	7 (18) 4 (13)	4 (11) 2 (7)
NSTS	2013	Crush vs. culotte	424	65 ± 10 65 ± 11	149 (71) 154 (71)	176 (84) 159 (74)	130 (62) 129 (60)	28 (13) 31 (15)	NA
NBS	2013	Provisional vs crush	404	63 ± 10 63 ± 10	154 (76) 158 (78)	158 (78) 146 (72)	109 (54) 117 (58)	26 (13) 24 (12)	NA
Ruiz et al.	2013	Provisional vs. T/TAP	69	63 ± 13 64 ± 13	28 (85) 28 (78)	17 (51) 23 (64)	22 (67) 26 (72)	15 (45) 12 (33)	NA
DKCRUSH-III	2015	DK crush vs. culotte	419	64 ± 10 63 ± 9	162 (77) 167 (80)	87 (41) 88 (42)	148 (71) 128 (61)	67 (32) 63 (30)	NA
BBK I	2015	Provisional vs. T/TAP	202	67 ± 10 67 ± 11	80 (79) 79 (78)	NA	93 (92) 90 (89)	26 (26) 19 (19)	19 (19) 21 (21)
TRYTON	2015	Dedicated vs. provisional	704	65 ± 11 65 ± 9	255 (72) 256 (73)	260 (74) 266 (77)	260 (73) 256 (74)	85 (24) 98 (28)	105 (30) 131 (38)
PERFECT	2015	Crush vs. provisional	419	61 ± 9 61 ± 9	160 (75) 155 (75)	132 (62) 118 (57)	118 (55) 114 (55)	55 (26) 60 (29)	9 (4) 9 (4)
BBK II	2016	Culotte vs T/TAP	300	66 ± 11 69 ± 10	107 (71) 114 (76)	NA	132 (88) 128 (85)	41 (27) 42 (28)	24 (16) 32 (21)
EBC TWO	2016	Provisional vs. culotte	200	63 ± 11 64 ± 12	87 (85) 76 (78)	72 (70) 70 (70)	65 (63) 66 (68)	26 (25) 30 (31)	40 (39) 40 (41)
SMART	2016	Provisional vs. T/TAP	258	62 ± 10 62 ± 10	105 (82) 108 (83)	16 (13) 17 (13)	70 (55) 75 (58)	37 (29) 33 (25)	7 (6) 5 (4)
Zhang et al.	2016	Provisional vs. culotte	104	65 ± 11 64 ± 7	48 (92) 43 (83)	6 (12) 6 (12)	35 (67) 33 (64)	10 (19) 11 (21)	12 (23) 10 (19)
Zheng et al.	2016	Crush vs. culotte	300	63 ± 8 64 ± 9	109 (73) 111 (74)	114 (76) 105 (70)	106 (71) 109 (73)	33 (22) 37 (25)	NA
DKCRUSH-II	2017	DK crush vs. provisional	366	64 ± 11 65 ± 10	145 (79) 138 (76)	62 (34) 53 (30)	120 (65) 111 (61)	36 (20) 42 (23)	32 (17) 24 (14)
COBRA	2018	Dedicated vs. culotte	40	66 ± 9 64 ± 10	14 (70) 15 (75)	19 (95) 19 (95)	15 (75) 14 (70)	5 (25) 4 (20)	6 (30) 2 (10)
DKCRUSH-V	2019	Provisional vs. DK crush	482	64 ± 10 65 ± 9	188 (78) 199 (83)	115 (48) 114 (48)	156 (65) 175 (73)	62 (26) 69 (29)	51 (21) 52 (22)
POLBOS I	2020	Dedicated vs provisional	243	66 ± 11 66 ± 9	82 (68) 84 (68)	75 (63) 70 (57)	94 (78) 90 (73)	45 (38) 31 (25)	55 (46) 43 (35)
POLBOS II	2020	Dedicated vs provisional	202	67 ± 9 67 ± 9	78 (76) 75 (75)	85 (83) 81 (81)	86 (84) 81 (81)	45 (44) 32 (33)	44 (43) 48 (48)
NBBS IV	2020	Provisional vs. culotte	446	64 ± 12 63 ± 11	NA	178 (82) 184 (81)	152 (70) 149 (66)	36 (17) 35 (16)	NA

Prior PCI	Prior CABG	Family history	Current smoking	LVEF	Unstable angina	Multivessel disease	Calcification	Chronic total occlusion	Final kissing inflation	Procedural success	Inclusion complex lesion
NA	NA	NA	NA	59 ± 10 59 ± 9	11 (17) 4 (17)	35 (56) 9 (39)	NA	NA	57 (90) 18 (82)	58 (92) 17 (77)	No
NA	NA	NA	18 (38) 23 (52)	60 ± 11 55 ± 11	42 (89) 38 (86)	NA	NA	NA	28 (60) 34 (77)	44 (94) 43 (97)	No
17 (11) 18 (12)	NA	NA	98 (63) 99 (64)	63 ± 13 62 ± 11	109 (70) 108 (70)	NA	0 (0) 1 (1)	3 (2) 7 (4)	124 (76) 161 (100)	142 (91) 149 (96)	No
55 (31) 46 (27)	8 (5) 10 (6)	83 (47) 62 (36)	36 (20) 29 (17)	55 ± 9 57 ± 8	78 (44) 63 (47)	NA	NA	NA	163 (92) 156 (90)	160 (90) 158 (91)	No
25 (45) 21 (39)	3 (5) 3 (6)	NA	10 (18) 4 (7)	62 ± 10 60 ± 10	NA	10 (18) 13 (24)	NA	NA	NA	NA	No
42 (17) 40 (16)	NA	104 (42) 103 (41)	42 (17) 43 (17)	56 ± 6 57 ± 6	NA	78 (31) 67 (27)	21 (8) 28 (11)	NA	72 (29) 189 (76)	235 (94) 234 (94)	No
13 (24) 13 (24)	5 (9) 4 (7)	NA	16 (30) 13 (24)	56 ± 6 57 ± 6	23 (43) 22 (41)	NA	14 (26) 15 (28)	0 (0) 1 (2)	51 (94) 49 (91)	NA	Yes
NA	NA	NA	NA	59 ± 10 57 ± 10	24 (96) 20 (77)	NA	NA	NA	NA	25 (100) 26 (100)	Yes
NA	NA	NA	NA	62 ± 10 64 ± 6	27 (71) 19 (63)	NA	NA	NA	38 (100) 25 (84)	NA	Yes
84 (40) 72 (34)	8 (4) 11 (5)	118 (57) 134 (62)	42 (20) 58 (27)	57 ± 11 57 ± 12	43 (21) 54 (26)	NA	NA	NA	177 (85) 197 (92)	205 (98) 210 (98)	No
51 (25) 51 (25)	8 (4) 6 (3)	117 (58) 109 (54)	NA	NA	65 (32) 69 (34)	NA	121 (60) 95 (47)	NA	65 (32) 150 (74)	196 (97) 190 (94)	No
7 (21) 9 (25)	2 (6) 0 (0)	NA	20 (61) 18 (50)	NA	NA	23 (70) 18 (50)	NA	NA	14 (42) 23 (64)	34 (100) 34 (94)	No
NA	NA	NA	NA	NA	NA	149 (71) 145 (70)	NA	NA	209 (99) 208 (99)	204 (97) 208 (99)	No
45 (45) 52 (52)	4 (4) 3 (3)	NA	10 (10) 14 (14)	59 ± 12 61 ± 12	NA	66 (65) 75 (74)	NA	NA	101 (100) 101 (100)	NA	Yes
135 (38) 146 (42)	9 (3) 7 (2)	NA	62 (18) 53 (15)	58 ± 10 58 ± 10	71 (20) 69 (20)	116 (33) 132 (38)	58 (16) 78 (22)	NA	305 (86) 297 (85)	283 (80) 246 (71)	No
NA	NA	30 (14) 26 (13)	54 (25) 67 (33)	60 ± 7 60 ± 7	74 (35) 65 (31)	NA	NA	NA	NA	NA	No
57 (38) 48 (32)	9 (6) 10 (7)	61 (41) 59 (39)	17 (11) 17 (11)	56 ± 7 57 ± 6	NA	129 (86) 135 (90)	NA	NA	150 (100) 150 (100)	150 (100) 148 (99)	No
41 (40) 40 (41)	NA	49 (48) 48 (49)	58 (56) 49 (50)	NA	NA	24 (23) 32 (33)	20 (19) 17 (17)	NA	97 (94) 93 (96)	100 (97) 95 (98)	Yes
14 (11) 9 (7)	0 (0) 1 (1)	17 (13) 19 (15)	33 (26) 23 (18)	61 ± 7 59 ± 10	26 (20) 31 (23)	NA	NA	NA	33 (26) 89 (69)	NA	No
13 (25) 12 (23)	0 (0) 0 (0)	NA	31 (60) 27 (52)	NA	25 (48) 28 (54	33 (64) 38 (73)	5 (10) 3 (6)	4 (8) 8 (15)	43 (83) 48 (92)	48 (92) 51 (98)	Yes
40 (27) 34 (23)	NA	45 (30) 52 (35)	58 (39) 67 (45)	NA	124 (83) 129 (86)	NA	NA	NA	107 (71) 129 (86)	145 (97) 148 (99)	No
39 (21) 38 (21)	0 (0) 1 (1)	NA	NA	NA	123 (67) 125 (69)	127 (70) 120 (65)	NA	NA	183 (100) 144 (79)	183 (100) 180 (99)	Yes
8 (40) 4 (20)	0 (0) 0 (0)	NA	5 (25) 4 (20)	67 ± 10 68 ± 11	4 (20) 4 (20)	NA	11 (55) 8 (40)	NA	20 (100) 20 (100)	18 (90) 20 (100)	No
43 (18) 33 (14)	2 (1) 2 (1)	NA	78 (32) 82 (34)	60 ± 9 59 ± 9	180 (74) 168 (70)	216 (89) 211 (88)	96 (40) 89 (37)	30 (12) 29 (12)	191 (80) 239 (99)	235 (97) 236 (98)	Yes
59 (49) 59 (48)	8 (7) 6 (5)	NA	26 (22) 31 (25)	NA	NA	NA	NA	NA	37 (31) 61 (50)	119 (99) 121 (98)	No
53 (52) 57 (57)	13 (13) 16 (16)	NA	21 (21) 26 (26)	NA	NA	74 (73) 68 (68)	NA	NA	34 (33) 49 (49)	101 (99) 99 (99)	No
77 (36) 76 (34)	8 (4) 4 (2)	108 (51) 107 (47)	41 (19) 48 (21)	57 ± 6 56 ± 7	28 (13) 38 (17)	NA	NA	NA	79 (36) 208 (91)	212 (98) 226 (99)	No

*T*/*TAP* T-stenting/T-stenting and protrusion, *DK* double kissing, *MI* myocardial infarction, *PCI* percutaneous coronary intervention, *CABG* coronary artery bypass graft, *LVEF* left-ventricular ejection fractions

### Network meta-analysis

**Fig. 2:**
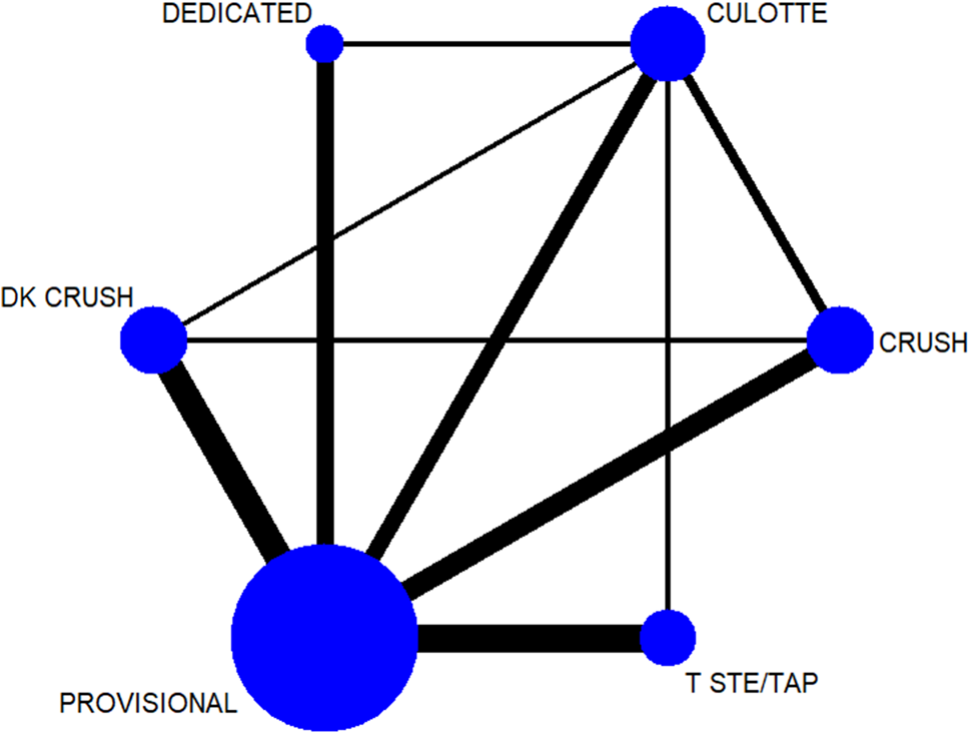
Network plot. *DK* double kissing, *TSTE*/*TAP* T-stenting/T-stenting and protrusion

**Fig. 3:**
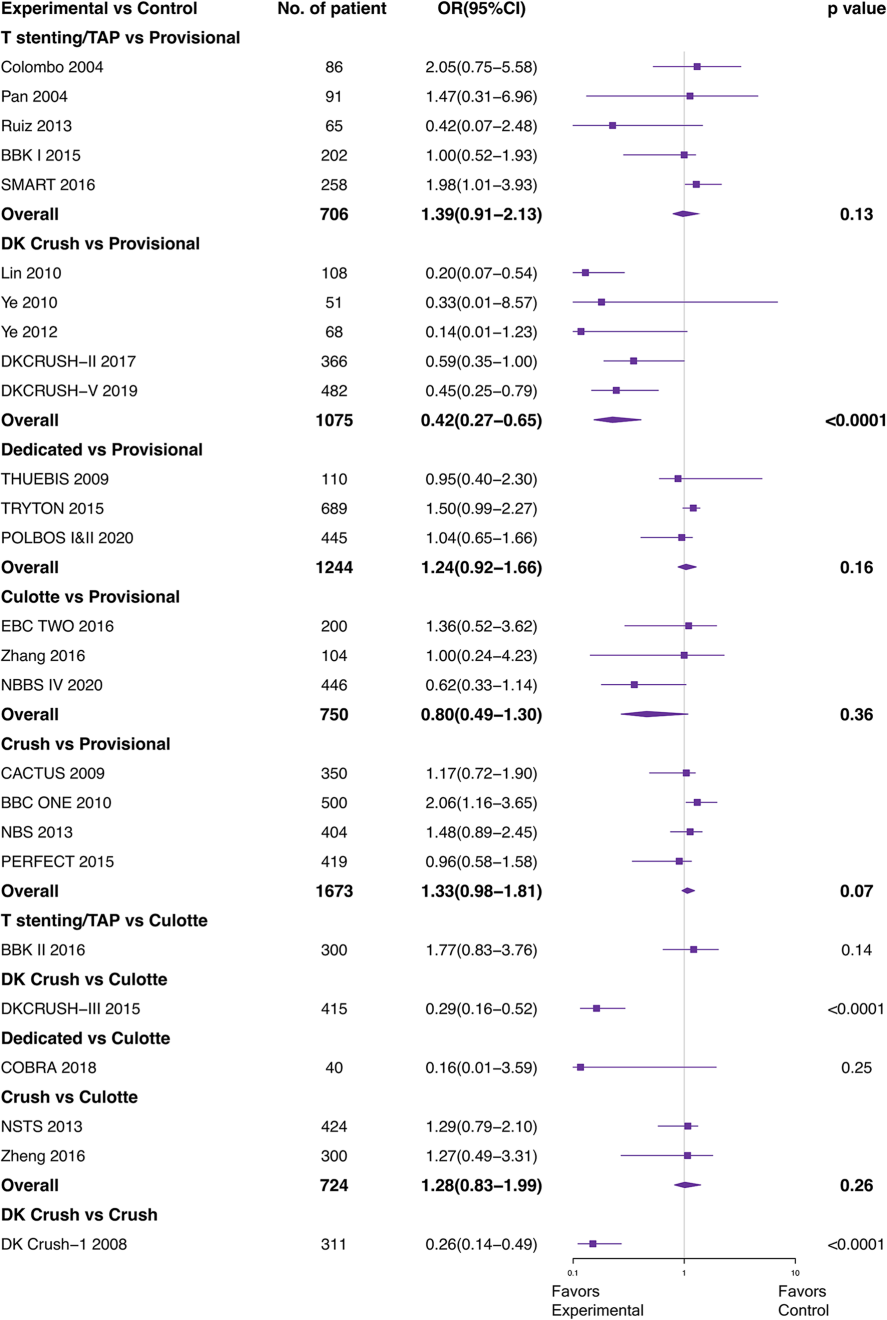
Direct pair-wise comparison for MACEs of 26 trails. *TAP* T-stenting and protrusion, *DK* double kissing

Figure 2 shows the network diagram design. The results of direct pair-wise comparison and combined ORs for MACEs are shown in Fig. 3. Compared with other stent strategies, DK crush had a lower incidence of MACEs: OR versus provisional 0.42 (95% CI 0.27–0.65); vs. culotte 0.29 (95% CI 0.16–0.52) and vs. crush 0.26 (95% CI 0.14–0.49). This result demonstrated that efficacy favors DK crush over provisional, culotte, and crush using direct comparison, arising from 7 studies. No major heterogeneity was observed with studies (*I*^*2*^ < 50%).

Table 2 summarizes the results of the multiple-treatment meta-analysis. DK crush was significantly more superior to other 5 stent techniques in MACEs: OR vs. provisional 0.40 (95% CI 0.28–0.55); vs. culotte 0.40 (95% CI 0.26–0.60). This benefit was driven by a lower incidence of cardiac death (crush vs. OR 2.65, 95% CI 1.20–6.78), TLR (culotte vs. OR 3.76, 95% CI 1.13–12.32), and ST (OR vs. provisional 0.35, 95% CI 0.23–0.53). Of note, DK crush was not significantly more efficacious than other strategies in reducing MI. And, there were no significant differences in all endpoints among the other 5 stent technology.

**Table 2:**
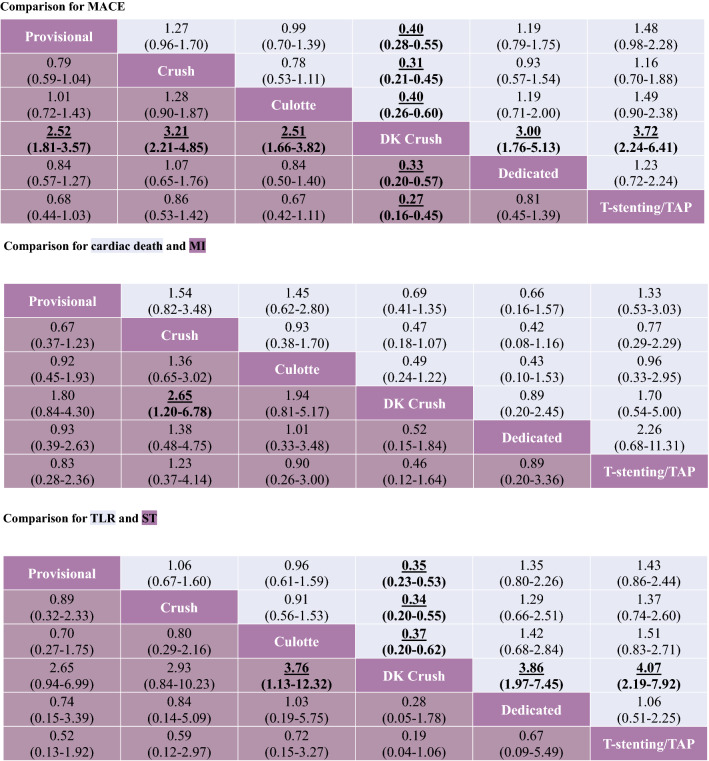
Network meta-analysis for primary and secondary endpoints

Results are the ORs in the column-defining treatment compared with the ORs in the row-defining treatment. For outcomes, ORs higher than 1 favor the row-defining treatment. Significant results are in bold and underscored

*TAP* T-stenting and protrusion, *DK* double kissing, *MACE* major adverse cardiovascular event, *MI* myocardial infarction, *TLR* target-lesion revascularization, *ST* stent thrombosis

**Fig. 4:**
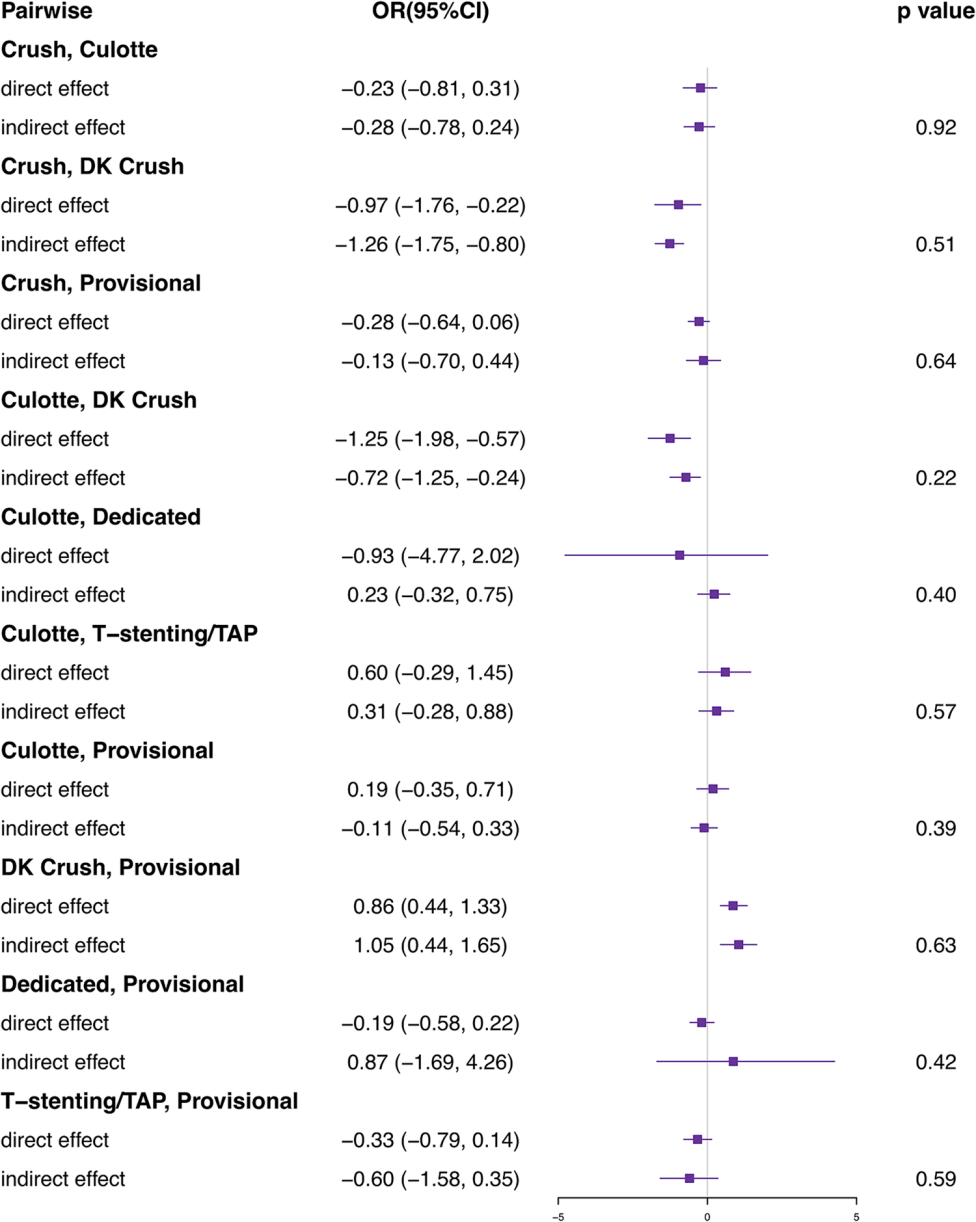
Node-splitting analysis. *DK* double kissing, *TAP* T-stenting and protrusion. ORs higher than 1 favor the prior treatment. *P* value which is greater than 0.05 is considered no inconsistency

Figure 4 shows the estimation of direct and network effects. It has demonstrated that there was no inconsistency (Bayesian *p* > 0.05) between all the pair-wise comparisons for MACEs.

**Fig. 5:**
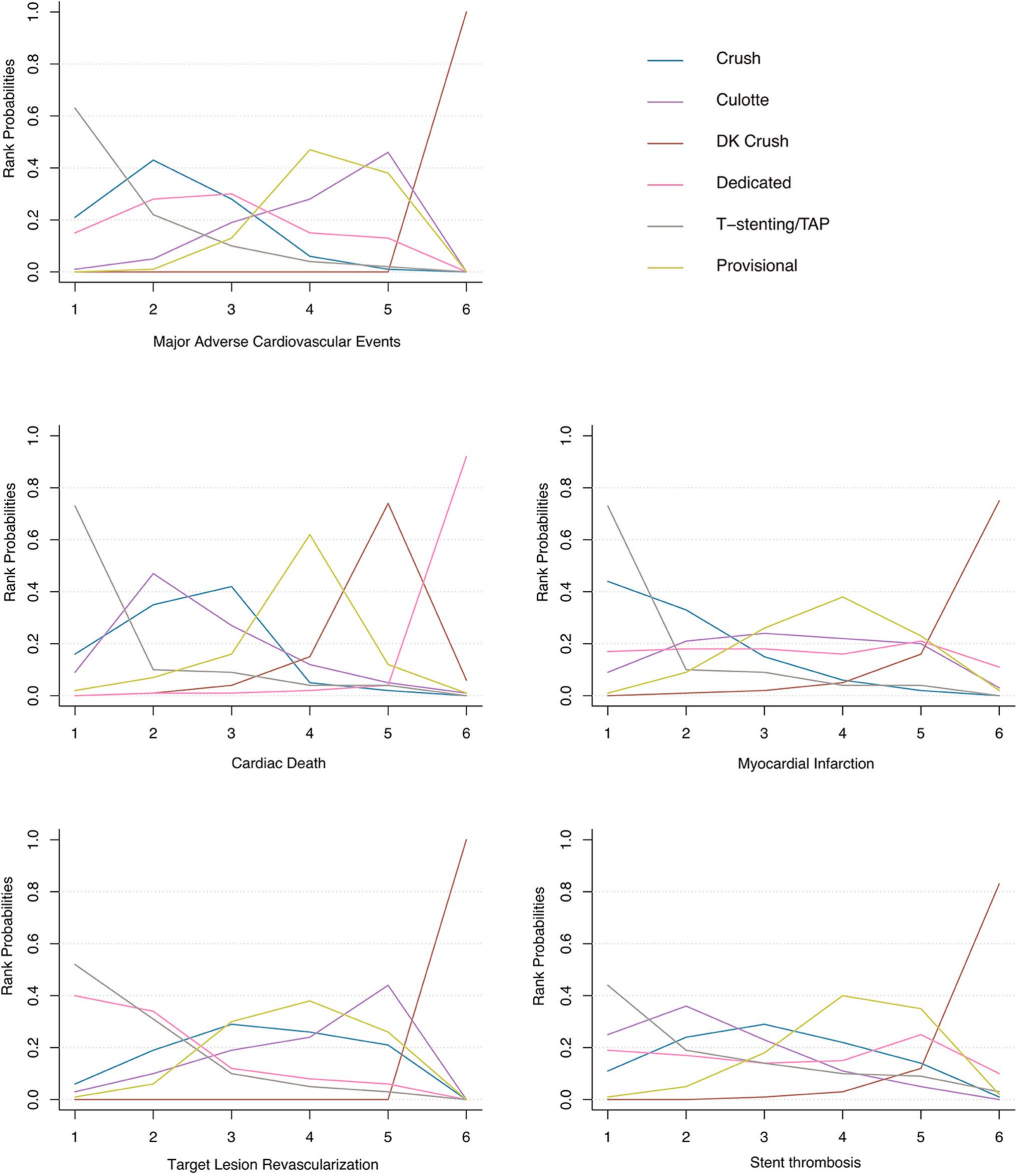
Rank probability analysis for primary and secondary endpoints. *DK* double kissing, *TAP* T-stenting and protrusion. Rank 1 is identified as the treatment with the highest incidence of each endpoint events and Rank 6 is identified as the treatment with the lowest incidence of each endpoint event

The distribution of probabilities of each stent technique being ranked at each of the possible 6 positions is shown in Fig. 5. DK crush ranked the most effective treatment for MACEs (100%), MI (75%), ST (83%), and TLR (100%). And for cardiac death, a dedicated stent was most likely to be the best treatment (92%), followed by DK crush (6%). T-stenting/TAP, and crush ranked the most ineffective treatment for all outcomes.

### Sensitivity analysis

Including only true bifurcation trails, DK crush was associated with lower incidence MACEs (*n* = 20; OR vs. provisional 0.37, 95% CI 0.24–0.55). After excluding studies of high-risk bias, DK crush was notably superior to other 5 treatments (OR vs. provisional 0.40, 95% CI 0.26–0.58). However, we only observed DK crush over crush in studies including only low-risk bias (crush vs. OR 2.60, 95% CI 1.06–6.61). Supplemental Table S7 and S8 show the sensitivity analysis. Supplemental Figure S1 displays that DK crush ranked the highest likelihood of being the best treatment to reduce MACEs in the sensitivity analysis.

### Complex lesion analysis

**Fig. 6:**
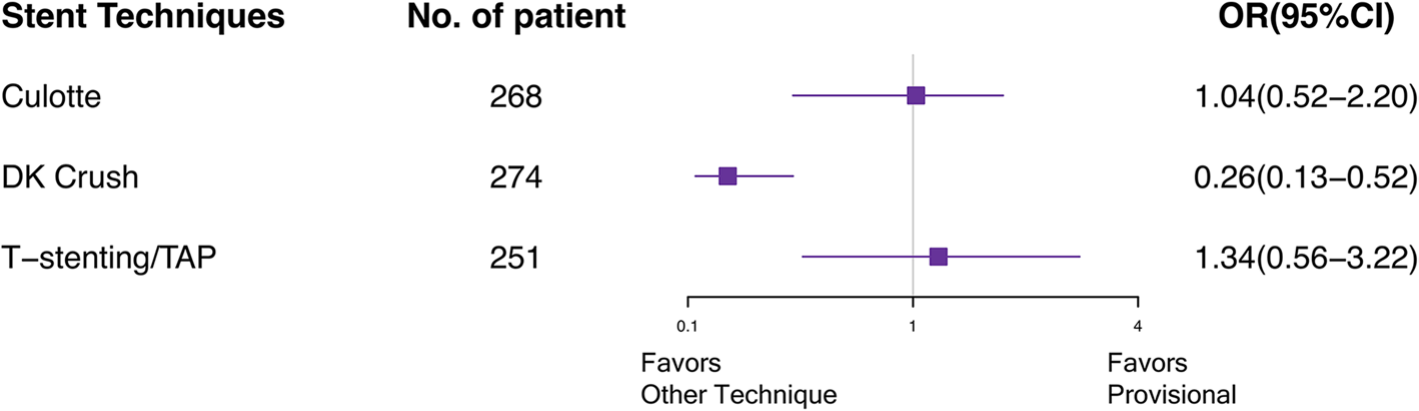
Forest plot for MACEs in patients with complex bifurcation lesions defined by DEFINITION criterion. *MACE* major adverse cardiovascular event, *DK* double kissing, *TAP* T-stenting and protrusion

**Fig. 7:**
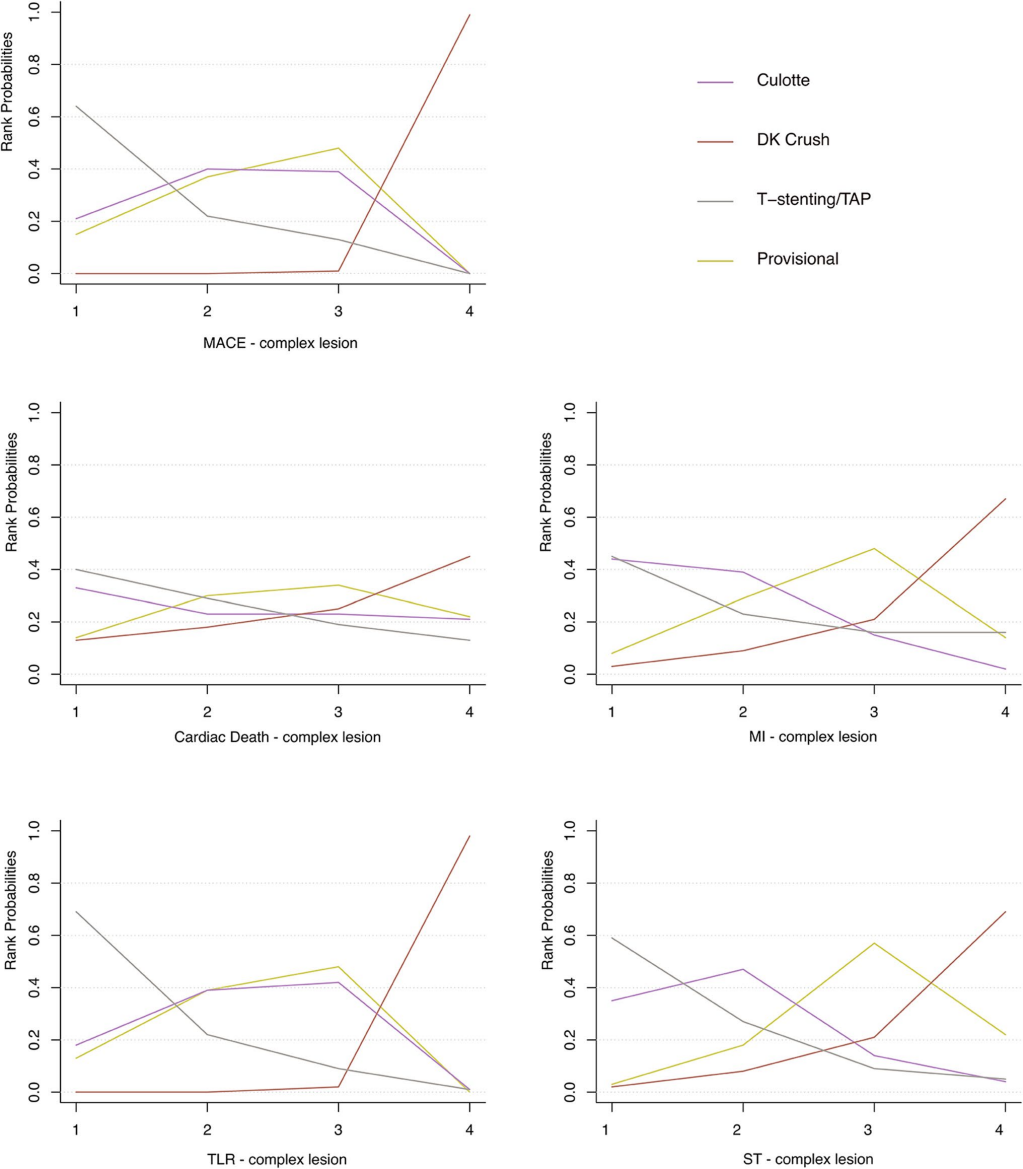
Rank probability analysis in patients with complex bifurcation lesions. *DK* double kissing, *TAP* T-stenting and protrusion, *MACE* major adverse cardiovascular event, *MI* myocardial infarction, *TLR* target-lesion revascularization, *ST* stent thrombosis. Rank 1 is identified as the treatment with the highest incidence of each endpoint events and Rank 4 is identified as the treatment with the lowest incidence of each endpoint events

The network meta-analysis of inclusion complex bifurcation lesion defined by The DEFINITION Study is shown in Table 3, [6]. The Forest plot in Fig. 6 showed that the benefit of 2-stent strategies was observed in complex lesions. In the treatment of complex bifurcation lesion, DK crush was notably more efficacious than provisional, culotte, and T-stenting/TAP in MACEs (OR vs. provisional 0.26, 95% CI 0.13–0.52) and TLR (OR vs. provisional 0.24, 95% CI 0.10–0.58). Rank probabilities are shown in Fig. 7. DK crush was most likely to be the best strategy of a complex lesion in all outcomes.

**Table 3:**
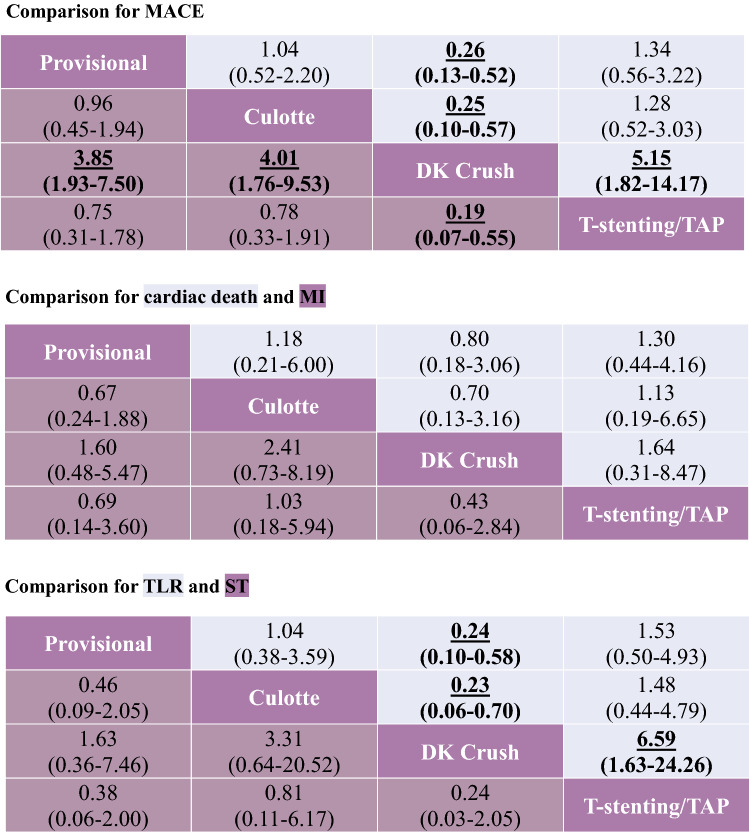
Network meta-analysis for primary and secondary endpoints in patients with complex bifurcation lesions defined by DEFINITION criterion

Results are the ORs in the column-defining treatment compared with the ORs in the row-defining treatment. For outcomes, ORs higher than 1 favor the row-defining treatment. Significant results are in bold and underscored

*TAP* T-stenting and protrusion, *DK* double kissing, *MACE* major adverse cardiovascular event, *MI* myocardial infarction, *TLR* target-lesion revascularization, *ST* stent thrombosis

## Discussion

Our analysis was based on 26 studies including 7257 individuals randomly assigned to 6 different stent techniques. Our findings might help to choose among stent techniques for treatment of coronary bifurcation lesions: 1) DK crush was significantly more superior than other 5 stent techniques in reducing MACE, cardiac death, TLR, and ST; 2) In patients with complex bifurcation lesion, DK crush was notably more efficacious than provisional, culotte, and T-stenting/TAP in reducing MACE and TLR; 3) DK crush was most likely to be the best treatment of coronary bifurcation lesion using rank probability analysis.

### Current guidelines prefer provisional strategy as the optimal treatment

These results are not in accordance with some current guidelines recommendations. The first principle of bifurcation stent placement is to keep the strategy simple and safe while understanding and respecting the original bifurcation anatomy. The European Bifurcation Club (EBC) recommends a provisional single-stent strategy as the optimal approach in the majority of cases [3]. Most randomized trails comparing 1-stent with 2-stent show that there is no benefit to implanting 2 stents with no relation to lesion type [7–9]. The provisional strategy describes PCI where using a single main vessel (MV) stent; the wired SB is not treated, or is treated only with balloon inflation. The EBC recommends that proximal optimization technique (POT) should be performed routinely during the bifurcation procedure as it reduces the risk of SB occlusion due to carina shift and facilitates SB access after MV stent implantation. While the decision of which technique to use in an individual lesion remains depending on the operator and the lesion, current guidelines prefer 1-stent strategy as it optimizes the flow and function of a bifurcation following PCIs and limits the number of stents which should be well apposed and expanded with limited overlap [3].

### DK crush has more advantages in some special bifurcation lesions

Although the EBC recommend provisional stent as the bifurcation PCIs of choice in the majority of bifurcation lesions, several factors may lead operators to adopt a 2-stent strategy. These factors consist of the importance of the SB and the risk of SB occlusion if a provisional stent was performed. Factors that operators tend to choose 2-stent strategy include true bifurcation lesions (Medina: 1,1,1; 1,0,1; or 0,1,1), SB diameter > 2.5 mm, length of SB lesion > 10–20 mm beyond SB ostium, and predicted adverse re-crossing after MV stent placement in the case of SB occlusion.

In the DKCRUSH-V trial performed by Chen et al. [5], DK crush had lower incidence of TLR and TVR on unprotected left main (LM) bifurcation lesions compared with provisional strategy. Unlike the EBC, based on this RCT, the European Society of Cardiology (ESC) and European Association for Cardio-Thoracic Surgery (EACTS) recommend the DK crush technique in true bifurcation lesions of the LM compared with the provisional technique.

DK crush, an iteration and modification of the classical crush technique for coronary bifurcation diseases, first described by Zhang et al. [10] The major difference between classic and DK crush is employing the first kissing balloon inflation (KBI) followed by the balloon crush of the SB stent implantation, leaving only one layer of metal struts at the ostial SB with minimal deformation, which furthers the second KBI after MV stenting. Compared with the classic crush technique, DK crush is superior in the rate of final KBI success and repeat intervention [4, 11].

Compared with other stent strategies, DK crush is associated with a higher rate of final KBI. Contrary to the 1-stent method, there is strong evidence to support the efficacy of final KBI in the 2-stent technique [12, 13]. Unsuccessful final KBI is associated with a significantly higher rate of MACE, TLR, and ST. Therefore, systematic FKB might be causally related to the better result of the DK crush over other stent techniques.

### DK crush is more superior in complex lesions

The MEDINA classification for bifurcation lesion was the most frequent classification, which does not provide adequate information about the true complexity of a given bifurcation lesion due to the lack of lesion specificity and clinical variables [14]. In the previous studies, using lesion complexity as a parameter may lead to different stent treatment; as a result, the final clinical outcome might be different.

The DEFINITION classification, first described by Chen et al., establishes the criteria differentiating simplex from complex bifurcation lesions. In summary, a complex bifurcation lesion is defined as any one of the main criteria (SB lesion length ≥ 10 mm with diameter stenosis (DS) of SB ≥ 70% for distal LM disease or DS of SB ≥ 90% for non-LM bifurcation lesions) plus any two minor criterion [moderate-to-severe calcification, multiple vessel lesions, bifurcation angle < 45° or > 70°, MV reference vessel diameter (RVD) < 2.5 mm, thrombus-containing lesions, and MV lesion length ≥ 25 mm] by visual estimation [6]. The new standard proposed by this study can distinguish between complex bifurcation lesions and simple bifurcation lesions and patients with complex bifurcation had very poor clinical prognosis [1-year follow-up MACE 16.8% vs. 8.9%, hazard ratio (HR): 0.72, 95% CI 0.51–0.93, *p* < 0.001]. In the present DEFINITION II trial by Zhang, et al., they demonstrated that a planned routine 2-stent strategy (mainly DK crush technique) reduced target-lesion failure (TLF) at 1 year compared with provisional stenting in patients with DEFINITION criteria-defined complex bifurcation lesions (77.8% of 2-stent strategy: DK crush, 6.1% vs. 11.4%, HR: 0.52, 95% CI 0.30–0.90, *p* = 0.019) [15]. Compared with the provisional stenting strategy, the systematic 2-stent approach, especially DK crush, improved the clinical outcomes significantly in patients with complex bifurcation lesions defined by the DEFINITION criterion.

## Limitation

Due to the characteristic of meta-analysis, we were unable to collect detailed information of all patients in these 26 trials. The endpoints defined by each study were not completely consistent, which may lead to heterogeneity. However, we considered that this only caused minor differences because of the consistent test across the trials. Our analysis was incapable of proving that DK crush has a significant advantage in reducing the incidence of MI. Moreover, we were powerless to reveal the superiority of DK crush of patients with complex bifurcation lesions in cardiac death, MI, and ST. Finally, because of the lack of individual information, we may not include all patients with complex bifurcation lesions defined by DEFINITION criteria. More randomized controlled trials are needed to compare the efficacy of different stenting strategies in coronary bifurcation disease. Further study is urgently warranted to prove the superiority of DK crush in patients with complex lesions.

## Conclusion

In our network meta-analysis, DK crush had a lower incidence of MACEs compared with other stenting strategies. This benefit was driven by a lower rate of cardiac death, TLR, and ST. Of note, DK crush was significantly more efficacious than provisional, culotte, and T-stenting/TAP in MACEs in patients with complex bifurcation lesions defined by the DEFINITION criterion.

## Supplementary Information

Below is the link to the electronic supplementary material.

Supplementary file1 (DOCX 643 KB)

## Abbreviations

CBD: Coronary bifurcation disease
PCI: Percutaneous coronary intervention
RCT: Randomized controlled trial
MACE: Major adverse cardiovascular event
MI: Myocardial infarction
TLR: Target lesion revascularization
TVR: Target vessel revascularization
ST: Stent thrombosis
DK: Double kissing
TAP: T-stenting and protrusion
SB: Side branch
MB: Main branch
CTO: Chronic total occlusion
PRISMA: Preferred reporting items for systematic reviews and meta-analyses
CABG: Coronary artery bypass graft
ARC: The academic research consortium
OR: Odds ratio
CI: Confidence interval
SUCRA: Surface under the cumulative ranking curve
EBC: The European bifurcation club
MV: Main vessel
POT: Proximal optimization technique
LM: Left main
ESC: The European society of cardiology
EACTS: European association for cardio-thoracic surgery
FKB: Final kissing balloon
KBI: Kissing balloon inflation
DS: Diameter stenosis
RVD: Reference vessel diameter
HR: Hazard ratio
TLF: Target lesion failure
