# Supplementary material for: Stenting techniques for coronary bifurcation disease: a systematic review and network meta-analysis demonstrates superiority of double-kissing crush in complex lesions
Source: Clin Res Cardiol. 2021 Dec 4;111(7):761–75. doi: 10.1007/s00392-021-01979-9 (PMC9242927; doi:10.1007/s00392-021-01979-9)
Supplement: Supplementary file 1 — Supplementary file1 (DOCX 643 KB) [file 392_2021_1979_MOESM1_ESM.docx]

**Stenting Techniques for Coronary Bifurcation Disease: A Systematic Review and Network Meta-Analysis Demonstrates Superiority of Double-Kissing Crush in Complex Lesions**

Rui Wang¹, MD, Yaodong Ding¹, MD, Jiaxin Yang¹, MD, Kexin Wang¹, MD, Wen Gao^2^, MD. Zhe Fang^3^, MD, Yujie Zhou¹, MD, Hailong Ge¹*, MD

*Correspondence: gehailong@126.com

¹Department of Cardiology, Beijing Anzhen Hospital, Capital Medical University, Beijing, 100029, PR China

^2^Department of Cardiology, Bayannaoer City Hospital, Inner Mongolia, 015002, PR China

^3^Department of Cardiology, Beijing Daxing District People's Hospital, Capital Medical University Daxing Teaching Hospital, Capital Medical University, Beijing, 102699, PR China

**Supplemental Table S1: Research strategies and keywords**

| **Database** | **Research strategy** | **Number** |
| --- | --- | --- |
| **Pubmed*** | ((bifurcation) AND (coronary stenosis[MeSH Terms])) AND (stents[MeSH Terms]) | **66** |
|  | ((bifurcation) AND (coronary stenosis[MeSH Terms])) AND (stents[MeSH Terms] OR Percutaneous Coronary Intervention[MeSH Terms]) | **70** |
|  | (bifurcation treatment) AND coronary | **189** |
|  | ((stents [MeSH Terms]) AND coronary stenosis [MeSH Terms]) AND (Major adverse cardiovascular events OR MACE) AND bifurcation | **30** |
|  | ((stents [MeSH Terms] OR Percutaneous Coronary Intervention [Mesh Terms]) AND coronary stenosis [MeSH Terms]) AND (Major adverse cardiovascular events OR MACE) AND bifurcation | **31** |
| **Embase** | ('bifurcation'/exp OR bifurcation) AND ('coronary artery obstruction'/exp OR 'coronary artery obstruction') AND ('cardiovascular stent'/exp OR 'cardiovascular stent')  AND ('clinical trial'/de OR 'controlled clinical trial'/de OR 'controlled study'/de OR 'randomized controlled trial'/de OR 'randomized controlled trial (topic)'/de) | **116** |
|  | 'bifurcation stent'/exp OR 'bifurcation stent' AND 'coronary artery obstruction'/exp OR 'coronary artery obstruction'  AND ('clinical trial'/de OR 'controlled clinical trial'/de OR 'controlled study'/de OR 'randomized controlled trial'/de OR 'randomized controlled trial (topic)'/de) | **9** |
|  | 'bifurcation'/exp OR 'bifurcation' AND 'coronary artery obstruction'/exp OR 'coronary artery obstruction' AND 'percutaneous coronary intervention'/exp OR 'percutaneous coronary intervention'  AND ('clinical trial'/de OR 'controlled clinical trial'/de OR 'controlled study'/de OR 'randomized controlled trial'/de OR 'randomized controlled trial (topic)'/de) | **122** |
| **Web of Science** | (coronary stenosis) AND (stents OR Percutaneous Coronary Intervention) AND (bifurcation) | **427** |
|  | ("bifurcation treatment") AND (coronary) | **38** |
|  | (coronary stenosis) AND ("Major adverse cardiovascular events" OR MACE) AND (bifurcation) AND (stents OR Percutaneous Coronary Intervention) | **96** |
| **Total articles** | | **1194** |

***Strategy from Pubmed: Refined by: ARTICLE TYPE (Randomized Controlled Trial)**

**Supplemental Table S2: PRISMA NMA Checklist of Items to Include When Reporting A Systematic Review Involving a Network Meta-analysis**

| **Section/Topic** | **Item** | **Checklist Item** | **Reported** |
| --- | --- | --- | --- |
|  | **#** |  | **on Page #** |
| **TITLE** |  |  |  |
|  |  |  |  |
| Title | 1 | Identify the report as a systematic review *incorporating a* | 1 |
|  |  | *network meta-analysis (or related form of meta-analysis).* |  |
| **ABSTRACT** |  |  |  |
| Structured | 2 | Provide a structured summary including, as applicable: |  |
| summary |  | **Background:** main objectives | 2 |
|  |  | **Methods:** data sources; study eligibility criteria, participants, | 2 |
|  |  | and interventions; study appraisal; and *synthesis methods,* |  |
|  |  | *such as network meta-analysis.* |  |
|  |  | **Results:** number of studies and participants identified; | 2 |
|  |  | summary estimates with corresponding confidence/credible |  |
|  |  | intervals; *treatment rankings may also be discussed. Authors* |  |
|  |  | *may choose to summarize pairwise comparisons against a* |  |
|  |  | *chosen treatment included in their analyses for brevity.* |  |
|  |  | **Discussion/Conclusions:** limitations; conclusions and | 3 |
|  |  | implications of findings. |  |
|  |  | **Other:** primary source of funding; systematic review | 5 |
|  |  | registration number with registry name. |  |
| **INTRODUCTION** |  |  |  |
|  |  |  |  |
| Rationale | 3 | Describe the rationale for the review in the context of what is | 4 |
|  |  | already known*, including mention of why a network meta-* |  |
|  |  | *analysis has been conducted.* |  |
| Objectives | 4 | Provide an explicit statement of questions being addressed, | 4 |
|  |  | with reference to participants, interventions, comparisons, |  |
|  |  | outcomes, and study design (PICOS). |  |
| **METHODS** |  |  |  |
|  |  |  |  |
| Protocol and | 5 | Indicate whether a review protocol exists and if and where it | 4-5 |
| registration |  | can be accessed (e.g., Web address); and, if available, provide |  |
|  |  | registration information, including registration number. |  |
| Eligibility criteria | 6 | Specify study characteristics (e.g., PICOS, length of follow-up) | 5-6 |
|  |  | and report characteristics (e.g., years considered, language, |  |
|  |  | publication status) used as criteria for eligibility, giving |  |
|  |  | rationale. *Clearly describe eligible treatments included in the* |  |
|  |  | *treatment network, and note whether any have been clustered* |  |
|  |  | *or merged into the same node (with justification).* |  |
| Information sources | 7 | Describe all information sources (e.g., databases with dates of | 5 |
|  |  | coverage, contact with study authors to identify additional |  |
|  |  | studies) in the search and date last searched. |  |
| Search | 8 | Present full electronic search strategy for at least one database, | Table S1 |
|  |  | including any limits used, such that it could be repeated. |  |
| Study selection | 9 | State the process for selecting studies (i.e., screening, | 5-6 |
|  |  | eligibility, included in systematic review, and, if applicable, |  |
|  |  |  |  |

|  |  | included in the meta-analysis). |  |  |
| --- | --- | --- | --- | --- |
|  |  |  |  |  |
| Data collection | 10 | Describe method of data extraction from reports (e.g., piloted |  | 5 |
| process |  | forms, independently, in duplicate) and any processes for |  |  |
|  |  | obtaining and confirming data from investigators. |  |  |
| Data items | 11 | List and define all variables for which data were sought (e.g., |  | 5 |
|  |  | PICOS, funding sources) and any assumptions and |  |  |
|  |  | simplifications made. |  |  |
| **Geometry of the** | **S1** | Describe methods used to explore the geometry of the |  | 6 |
| **network** |  | treatment network under study and potential biases related to it. |  |  |
|  |  | This should include how the evidence base has been |  |  |
|  |  | graphically summarized for presentation, and what |  |  |
|  |  | characteristics were compiled and used to describe the evidence |  |  |
|  |  | base to readers. |  |  |
| Risk of bias within | 12 | Describe methods used for assessing risk of bias of individual |  | 5 |
| individual studies |  | studies (including specification of whether this was done at the |  |  |
|  |  | study or outcome level), and how this information is to be used |  |  |
|  |  | in any data synthesis. |  |  |
| Summary measures | 13 | State the principal summary measures (e.g., risk ratio, |  | 5-6 |
|  |  | difference in means). *Also describe the use of additional* |  |  |
|  |  | *summary measures assessed, such as treatment rankings and* |  |  |
|  |  | *surface under the cumulative ranking curve (SUCRA) values,* |  |  |
|  |  | *as well as modified approaches used to present summary* |  |  |
|  |  | *findings from meta-analyses.* |  |  |
| Planned methods of | 14 | Describe the methods of handling data and combining results of |  | 6 |
| analysis |  | studies for each network meta-analysis. This should include, |  |  |
|  |  | but not be limited to: |  |  |
|  |  | • *Handling of multi-arm trials;* |  |  |
|  |  | • *Selection of variance structure;* |  |  |
|  |  | • *Selection of prior distributions in Bayesian analyses;* |  |  |
|  |  | *and* |  |  |
|  |  | •*Assessment of model fit.* |  |  |
| **Assessment of** | **S2** | Describe the statistical methods used to evaluate the agreement |  | 6 |
| **Inconsistency** |  | of direct and indirect evidence in the treatment network(s) |  |  |
|  |  | studied. Describe efforts taken to address its presence when |  |  |
|  |  | found. |  |  |
| Risk of bias across | 15 | Specify any assessment of risk of bias that may affect the | 7 | 5 |
| studies |  | cumulative evidence (e.g., publication bias, selective reporting |  |  |
|  |  | within studies). |  |  |
| Additional analyses | 16 | Describe methods of additional analyses if done, indicating |  | 6 |
|  |  | which were pre-specified. This may include, but not be limited |  |  |
|  |  | to, the following: |  |  |

• Sensitivity or subgroup analyses; • Meta-regression analyses;

• *Alternative formulations of the treatment network; and* • *Use of alternative prior distributions for Bayesian*

*analyses (if applicable).*

| **RESULTS†** |  |  |  |
| --- | --- | --- | --- |
|  |  |  |  |
| Study selection | 17 | Give numbers of studies screened, assessed for eligibility, and | 7, Figure 1 |
|  |  | included in the review, with reasons for exclusions at each |  |
|  |  | stage, ideally with a flow diagram. |  |
| **Presentation of** | **S3** | Provide a network graph of the included studies to enable | Figure 2 |
| **network structure** |  | visualization of the geometry of the treatment network. |  |
| **Summary of** | **S4** | Provide a brief overview of characteristics of the treatment | 7 |
| **network geometry** |  | network. This may include commentary on the abundance of |  |
|  |  | trials and randomized patients for the different interventions |  |
|  |  | and pairwise comparisons in the network, gaps of evidence in |  |
|  |  | the treatment network, and potential biases reflected by the |  |
|  |  | network structure. |  |
| Study | 18 | For each study, present characteristics for which data were | Table 1 |
| characteristics |  | extracted (e.g., study size, PICOS, follow-up period) and |  |
|  |  | provide the citations. |  |
| Risk of bias within | 19 | Present data on risk of bias of each study and, if available, any | Table S3 |
| studies |  | outcome level assessment. |  |
|  |  |  |  |
| Results of | 20 | For all outcomes considered (benefits or harms), present, for | 7, Figure 3 |
| individual studies |  | each study: 1) simple summary data for each intervention |  |
|  |  | group, and 2) effect estimates and confidence intervals. |  |
|  |  | *Modified approaches may be needed to deal with information* |  |
|  |  | *from larger networks.* |  |
| Synthesis of results | 21 | Present results of each meta-analysis done, including | 8, Table 2 |
|  |  | confidence/credible intervals. *In larger networks, authors may* |  |
|  |  | *focus on comparisons versus a particular comparator (e.g.* |  |
|  |  | *placebo or standard care), with full findings presented in an* |  |
|  |  | *appendix. League tables and forest plots may be considered to* |  |
|  |  | *summarize pairwise comparisons.* If additional summary |  |
|  |  | measures were explored (such as treatment rankings), these |  |
|  |  | should also be presented. |  |
| **Exploration for** | **S5** | Describe results from investigations of inconsistency. This may | 8, Figure 4 |
| **inconsistency** |  | include such information as measures of model fit to compare |  |
|  |  | consistency and inconsistency models, *P* values from statistical |  |
|  |  | tests, or summary of inconsistency estimates from different |  |
|  |  | parts of the treatment network. |  |
| Risk of bias across | 22 | Present results of any assessment of risk of bias across studies | 8, Table S7-8 |
| studies |  | for the evidence base being studied. |  |
|  |  |  |  |
| Results of | 23 | Give results of additional analyses, if done (e.g., sensitivity or | 9, Table 3 |
| additional analyses |  | subgroup analyses, meta-regression analyses*, alternative* |  |
|  |  | *network geometries studied, alternative choice of prior* |  |
|  |  | *distributions for Bayesian analyses,* and so forth). |  |
| **DISCUSSION** |  |  |  |
|  |  |  |  |
| Summary of | 24 | Summarize the main findings, including the strength of | 9 |
| evidence |  | evidence for each main outcome; consider their relevance to |  |
|  |  | key groups (e.g., healthcare providers, users, and policy- |  |
|  |  | makers). |  |
| Limitations | 25 | Discuss limitations at study and outcome level (e.g., risk of | 12 |
|  |  | bias), and at review level (e.g., incomplete retrieval of |  |
|  |  | identified research, reporting bias). *Comment on the validity of* |  |
|  |  | *the assumptions, such as transitivity and consistency. Comment* |  |

|  |  | *on any concerns regarding network geometry (e.g., avoidance* |  |  |
| --- | --- | --- | --- | --- |
|  |  | *of certain comparisons).* |  |  |
|  |  |  |  |  |
| Conclusions | 26 | Provide a general interpretation of the results in the context of |  | 12 |
|  |  | other evidence, and implications for future research. |  |  |
|  |  |  |  |  |
| **FUNDING** |  |  |  |  |
| Funding | 27 | Describe sources of funding for the systematic review and other |  | 13 |
|  |  | support (e.g., supply of data); role of funders for the systematic |  |  |
|  |  | review. This should also include information regarding whether |  |  |
|  |  | funding has been received from manufacturers of treatments in |  |  |

the network and/or whether some of the authors are content experts with professional conflicts of interest that could affect use of treatments in the network.

PICOS = population, intervention, comparators, outcomes, study design.

* Text in italics indicates wording specific to reporting of network meta-analyses that has been added to guidance from the PRISMA statement.

† Authors may wish to plan for use of appendices to present all relevant information in full detail for items in this section.

**Supplemental Table S3: Bias assessment**

| Study | Randomization Process | | Deviation from intended intervention | Missing data | Measurement of the outcome | Selection of the reported result | Risk of bias level |
| --- | --- | --- | --- | --- | --- | --- | --- |
|  | Random sequence generation | Allocation concealment |  |  |  |  |  |
| Colombo et al. |  |  |  |  |  |  |  |
| Pan et al. |  |  |  |  |  |  |  |
| DKCRUSH-1 |  |  |  |  |  |  |  |
| CACTUS |  |  |  |  |  |  |  |
| THUEBIS |  |  |  |  |  |  |  |
| BBC ONE |  |  |  |  |  |  |  |
| Lin et al. |  |  |  |  |  |  |  |
| Ye et al. |  |  |  |  |  |  |  |
| Ye et al. |  |  |  |  |  |  |  |
| NSTS |  |  |  |  |  |  |  |
| NBS |  |  |  |  |  |  |  |
| Ruiz et al. |  |  |  |  |  |  |  |
| DKCRUSH-III |  |  |  |  |  |  |  |
| BBK I |  |  |  |  |  |  |  |
| TRYTON |  |  |  |  |  |  |  |
| PERFECT |  |  |  |  |  |  |  |
| BBK II |  |  |  |  |  |  |  |
| EBC TWO |  |  |  |  |  |  |  |
| SMART |  |  |  |  |  |  |  |
| Zhang et al. |  |  |  |  |  |  |  |
| Zheng et al. |  |  |  |  |  |  |  |
| DKCRUSH-II |  |  |  |  |  |  |  |
| COBRA |  |  |  |  |  |  |  |
| DKCRUSH-V |  |  |  |  |  |  |  |
| POLBOS I&II |  |  |  |  |  |  |  |
| NBBS IV |  |  |  |  |  |  |  |

**Low risk of bias**

**Some concerns**

**High risk of bias**

**Supplemental Table S4: Endpoint and follow-up definition across the studies**

| Trial/First Author | Intervention | MACE Definition | Cardiac Death Definition | MI Definition | TVR/TLR/TVF/TLF Definition | DAPT | Intracoronary Imaging | Angiographic Follow-Up | Clinical Follow-Up |
| --- | --- | --- | --- | --- | --- | --- | --- | --- | --- |
| Colombo et al.  2004 | T-stenting vs. Provisional | Death, MI or TVR | NA | NA | TLR and TVR were defined as repeat revascularization driven by symptoms or laboratory testing and a stenosis≥50% within the treated vessel on follow-up angiography.  TVF was defined as presence of cardiac death, Q-wave or non-Q-wave MI, or TVR. | 3months | After the procedure and at follow-up | 6 months | 6 months |
| Pan et al.  2004 | Provisional vs. T-stenting | Cardiac death, MI or TVR | NA | A non-Q-wave MI was defined as an increase in creatine kinase level to >3 times the upper limit of the normal range. | NA | 12 months | IVUS in 41% of cases | 6 months | 6 months |
| DKCRUSH-I  2008 | Crush vs. DK Crush | Cardiac death, MI or TLR | NA | MI was defined as creatine kinase-MB (CK-MB) enzyme elevation ≥3 times the upper limit of the normal value, either with (Q wave MI) or without (non-Q wave MI), and new Q waves in at least two contiguous leads on electrocardiogram. | TLR was defined as repeat revascularization with a diameter stenosis ≥ 50% within the stent or in the 5 mm distal or proximal segments adjacent to the stent.  TVR was defined as repeat revascularization within the treated vessel. | 12 months | NA | 8 months | 8 months |
| CACTUS  2009 | Crush vs. Provisional | Cardiac death, MI or TVR | NA | Q-wave MI was defined as the development of new, pathological Q waves in 2 or more contiguous leads with postprocedure CK or CK-MB levels above normal. Non-Q-wave MI was defined as an elevation of postprocedural CK levels >2 times normal levels with elevated CK-MB in the absence of pathological Q waves. | NA | ≥6 months | IVUS in 6% of cases | 6 months | 6 months |
| THUEBIS  2009 | Dedicated vs. Provisional | Death, MI, ST, CABG or TLR | All deaths were considered cardiac unless otherwise documented. | The diagnosis of acute myocardial infarction (STEMI or NSTEMI) both periprocedurally and at follow-up, required an elevation of creatine kinase to levels twice those of the upper normal limit together with a rise in the creatine kinase-MB fraction, an elevation of troponin I and/or new ST-segment elevations or new Q-waves (ECG). The threshold used to define a positive troponin I test was 0.1 ng/mL. For CK, the manufacturer reported a lower limit of >2.8 μmol/s/L. | TLR was defined as either surgical or percutaneous reintervention driven by (1) significant (>50%) luminal diameter narrowing either within the stent or within the 5 mm proximal or distal to the MB or SB stent edge, (2) stent thrombosis, or (3) TLR-related CABG.  TVR was defined as revascularization by PCI or surgery within the target vessel encompassing the target lesion including TLR plus PCI target vessel-nontarget lesion plus CABG including target vessel, not related to the target lesion. | ≥6 months | NA | 6 months | 6 months |
| BBC ONE  2010 | Provisional vs. Crush | Death, MI, TVF | NA | Typical rise and fall of biochemical markers of myocardial necrosis with ischemic symptoms or ECG changes as per European Society of Cardiology/American College of Cardiology guidelines. For patients in the first 24 hours after PCI, CK ≥3 times the upper limit of normal was taken as the cutoff point for the diagnosis of myocardial infarction. For patients who already had a diagnosis of myocardial infarction on the current admission, CK rise to ≥50% of the previous value was used. | TVF comprised target-vessel revascularization by PCI or coronary artery bypass grafting of either the main vessel or side branch and/or target-vessel inadequacy (TIMI flow <3 in either the main vessel or side branch after appropriate vasodilators on a repeat angiogram, without attempted revascularization). | ≥9 months | NA | NO | 9 months |
| Lin et al.  2010 | Provisional vs. DK Crush | Cardiac death, MI, ST or TVR |  | Procedure-related MI was considered if CK-MB or troponin-I increased to more than three times the upper limit of normal (ULN). In the absence of a new Q wave, CK-MB at least 3×ULN was defined as a non-Q wave MI. Development of a new Q wave in two or more contiguous electro- cardiogram leads, with CK-MB at least 3×ULN, was defined as a new Q wave infarction. | TLR was defined as repeat revascularization with stenosis diameter at least 50% within the stent or in the adjacent segments 5mm distally or proximally to the stent. If separate stents were placed at either end of a target lesion, this counted as two interventions.  TVR was recognized as a repeat revascularization within the treated vessel. | 12 months | NA | 8months | 8 months |
| Ye et al.  2010 | DK Crush vs. Provisional | Cardiac death, MI, TLR and TVR | All deaths were considered to be of cardiac origin unless otherwise documented. | A non-Q-wave MI was defined as a rise of creatinine kinase-MB concentration to three times the upper limit of normal in the absence of pathological Q waves. | TLR was defined as a repeat revascularization for a stenosis greater than 50% in the target lesion of either the MB or SB.  TVR was defined as any repeat revascularization within the treated vessel. | ≥12 months | NA | 8 months | 8 months |
| Ye et al.  2012 | DK Crush vs. Provisional | Cardiac death, MI, TVR or TLR | All deaths were regarded as being of cardiac origin unless otherwise documented. | A non-Q wave myocardial infarction was defined as a creatine kinase (CK)-MB concentration increase three times the upper limit of the normal value in the absence of pathological Q waves. | TLR was defined as a repeated revascularization with a stenosis >50% in the target lesion in either the MV or SB.  TVR was defined as any repeat revascularization within the treated vessel, in the setting of symptomatic chest pain. | ≥12 months | Operator’s discretion | 8 months | 8 months |
| NSTS  FU 3 years  2013 | Crush vs. Culotte | Cardiac death, MI, ST or TVR | Death was considered cardiac unless other cause documented. | Nonprocedural MI was defined as a rise of biochemical markers exceeding the decision limit of MI (above the 99th percentile including <10% CV) associated with either typical symptoms and/or electrocardiographic changes. | TLR was defined as repeated revascularization by PCI or surgery of the target lesion.  TVR was defined as repeated revascularization by PCI or surgery of the target vessel. | 6 to 12 months | NA | 8 months | 5 years |
| NBS  FU 5 years  2013 | Provisional vs. Crush | Cardiac death, MI, TVR and ST | Death was considered cardiac unless other cause documented. | Non–Q-wave MI: a CK-MB mass or troponin-T/troponin-I increase to ≥3 URL combined with clinical signs of myocardial infarction, in the absence of pathological Q waves and not related to an interventional procedure.  Q-wave MI: a development of new pathological Q waves in 2 or more contiguous leads together with clinical signs of myocardial infarction (chest pain or increase in myocardial injury markers). | TLR was defined as repeated revascularization by PCI or surgery of the target lesion.  TVR was defined as repeated revascularization by PCI or surgery of the target vessel. | 6 to 12 months | NA | 8 months | 5 years |
| Ruiz et al.  2013 | Provisional vs. T-stenting | Cardiac death, MI or TVR | NA | MI defined as hospital admission with a diagnosis of acute coronary syndrome with or without ST segment elevation. | NA | 12 months | NA | 9 months | 9 months |
| DKCRUSH-III  FU 3 years  2015 | DK Crush vs. Culotte | Cardiac death, MI and/or TVR | All deaths were considered cardiac in origin unless noncardiac reasons were indicated. | MI was diagnosed if the plasma level of creatine kinase-myocardial band and/or troponin I/T increased to more than 3 times the upper normal limit in no fewer than 2 blood samples. | TLR and TVR were defined as any repeat revascularization (percutaneous coronary intervention or CABG) for target lesions and target vessels, respectively, in the presence of symptoms or objective signs of ischemia. | 12 months | IVUS in >70% of cases | 8 months | 3 years |
| BBK I  FU 5 years  2015 | Provisional vs. T-stenting | Death, MI and TLR | NA | MI was defined as the presence of new Q waves in two or more contiguous electrocardiographic leads or an elevation of creatine kinase or its MB isoenzyme to at least three times the upper limit of normal in two samples during hospitalization.  After discharge, the diagnosis of myocardial infarction was made according to the European Society of Cardiology/American College of Cardiology consensus document and based on new rise in troponin T ≥0.03 mg/L associated with either typical symptoms and/or typical ECG changes and/or typical angiographic findings. | TLR defined as coronary artery bypass surgery or repeat percutaneous angioplasty involving the stented segment and performed for symptoms or signs of ischaemia in the presence of angiographic restenosis. | 6 months | NA | 9 months | 5 years |
| TRYTON  2015 | Dedicated vs. Provisional | Death, MI, emergent CABG, clinically driven TLR | NA | Q-wave or non– Q-wave [3×upper limit of normal creatine kinase {CK}-MB]. | TVF defined as cardiac death, MI and clinically driven TVR. | ≥6 months | NA | 9 months | 9 months |
| PERFECT  2015 | Crush vs. Provisional | Death, MI and TVR. | Deaths were considered cardiac unless an unequivocal, noncardiac cause was established. | MI was defined as an increase in creatine kinase-myocardial band concentration to >3× the upper limit of the normal range, with ischemic symptoms or new ischemic electrocardiographic changes. | TLR was defined as repeat revascularization with PCI or coronary artery bypass surgery for restenosis of the entire segment involving the implanted stent and within 5 mm of the distal and proximal margins of the stent.  TVR was defined as any repeat revascularization in the treated vessel and was considered clinically driven when the treated vessels had at least 50% stenosis in the presence of ischemic signs or symptoms. | ≥12 months | IVUS in >95% of cases | 8 months | 12 months |
| BBK II  2016 | Culotte vs. TAP | Cardiac death, MI or TLR | NA | MI was defined as the presence of new Q waves in two or more contiguous electrocardiographic leads or an elevation of creatine kinase or its MB isoenzyme to at least three times the upper limit of normal in two samples during hospitalization.  After discharge, the diagnosis of myocardial infarction was made according to the European Society of Cardiology/American College of Cardiology consensus document and based on new rise in troponin T ≥0.03 mg/L associated with either typical symptoms and/or typical ECG changes and/or typical angiographic findings. | Clinically indicated TLR was defined as coronary artery bypass surgery or repeat PCI involving the stented segment and performed for symptoms or signs of ischemia in the presence of angiographic restenosis or for high grade (>70%) angiographic restenosis irrespective of the clinical presentation. TLF defined as a composite of death from cardiac causes, any myocardial infarction (not clearly attributable to a non-target vessel), or target lesion revascularization. | 6 months | NA | 9 months | 12 months |
| EBC TWO  2016 | Provisional vs. Culotte | Death, myocardial infarction or  target vessel failure | NA | Typical rise and fall of biochemical markers of myocardial necrosis with ischemic symptoms or ECG changes as per European Society of Cardiology/ American College of Cardiology guidelines.  Periprocedural MI is arbitrarily defined by the elevation of cTn values (>5×99th percentile URL) in patients with normal baseline values (≤99th percentile URL) or a rise of cTn values >20% if the baseline values are elevated and are stable or falling.  In addition, either (1) symptoms suggestive of myocardial ischemia, (2) new ischemic ECG changes, or (3) angiographic findings consistent with a procedural complication or (4) imaging demonstration of new loss of viable myocardium or new regional wall motion abnormality are required. | TVR comprised target vessel revascularization by PCI or coronary artery bypass graft of either the MV or the SB or target vessel inadequacy—<thrombolysis in myocardial infarction 3 flow in either MV or SB after appropriate vasodilators on a repeat angiogram, without attempted repeat intervention. | 12 months | NA | NO | 12 months |
| SMART-STRATEGY  FU 3 years  2016 | Provisional vs. T-stenting | Cardiac death, spontaneous MI, or TVR | All deaths were considered cardiac unless a definite noncardiac cause could be established. | Spontaneous MI was defined as elevated cardiac enzymes (troponin or myocardial band fraction of CK) greater than the upper limit of the normal that occurred along with ischemic symptoms or electrocardiography findings indicative of ischemia unrelated to the index procedure. | TVF: cardiac death, spontaneous MI, TVR. TVR: repeat revascularization of the target vessel by PCI or bypass graft surgery. TLR: repeat PCI of the lesion within 5 mm of stent deployment or bypass graft surgery of the target vessel. TBR: repeat revascularization with a stenosis diameter 50% within 5 mm proximal or distal to carina of bifurcation. | NA | 9 months | 9 months | 3 years |
| Zhang et al.  2016 | Provisional vs. Culotte | Cardiac death, MI, TLR/TVR and ST | NA | NQWMI was defined as a CK-MB or cTnT/cTnI that had increased to ≥3 times the upper limit of the normal range combined with clinical signs of MI, without new onset of pathological Q waves.  QWMI was defined as new development of pathological Q waves in two contiguous leads, together with clinical signs of MI (chest pain or increase in myocardial injury markers). | TLR/TVR was the repeat target lesion/vessel therapy either by PCI or by surgery. | 12 months | NA | 9 months | 9 months |
| Zheng et al.  2016 | Crush vs. Culotte | Cardiac death, MI, ST, and/ or TVR | According to the Academic Research Consortium definition. | According to the Academic Research Consortium definition. | According to the Academic Research Consortium definition. | ≥12 months | NA | 12 months | 12 months |
| DKCRUSH-II  FU 5 years  2017 | DK Crush vs. Provisional | Cardiac death, MI and TVR | All deaths were considered as cardiac in origin unless noncardiac reasons were indicated. | MI was diagnosed if the plasma level of creatine kinase (CK)-MB and troponin I/T increased to >3× the upper normal limit in no fewer than 2 blood samples. | TLR and TVR were defined as any repeat revascularization (percutaneous coronary intervention or coronary artery bypass graft) for target lesions and target vessels, respectively, in the presence of symptoms or objective signs of ischemia. | ≥12 months | IVUS in >46% of cases | 8 months | 5 years |
| COBRA  fu 5 years  2018 | Dedicated vs. Culotte | Cardiac death, MI, TLR, TVR, non-TVR, non-TLR and ST | NA | MI was defined as evidence of myocardial necrosis in a clinical setting consistent with myocardial ischemia. Typically, this included detection of rise in cardiac biomarkers with at least one value above the 99th percentile of the URL together with symptoms of ischemia, or pathognomonic electrocardiographic or imaging evidence of ischemia. By convention, increases of cardiac biomarkers above 3x the URL were used to define PCI-related MI. | TLR was only performed if there was significant angiographic restenosis (70% diameter stenosis anywhere within the target lesion) in combination with clear angina or a fractional flow reserve <0.80 in the MV or a SB subtending a large myocardial territory. | ≥12 months | NA | 9 months | 5 years |
| DKCRUSH-V  fu 3 years  2019 | Provisional vs. DK Crush | Cardiac death, target-vessel MI or clinically driven TLR | Death from cardiac causes was defined as any death without a clear noncardiac cause. | Protocol-defined periprocedural MI was defined as creatine kinase-myocardial band >10× the upper reference limit of the assay, or >5× upper reference limit plus: 1) new pathological Q waves in ≥2 contiguous leads or new left bundle branch block; 2) angiographically documented graft or coronary artery occlusion or new  severe stenosis with thrombosis; or 3) imaging evidence of new loss of viable myocardium or new regional wall motion abnormality.  Spontaneous MI (after 72h) was defined as a clinical syndrome consistent with MI with creatine kinase-myocardial band or troponin >1× upper reference limit and new ST-segment elevation or depression or other findings as previously mentioned. | Clinically driven TLR was defined as angina or ischemia referable to the target lesion requiring repeat PCI or CABG.  TLF was defined as the composite of cardiac death, target vessel MI [TVMI], or clinically driven TLR. | ≥12 months | IVUS in 42% of cases | 13 months | 3 years |
| POLBOS I&II  fu 4 years  2020 | Provisional vs. Dedicated | Cardiac death, MI and TLR | Cardiac death included death resulting from an acute MI, sudden cardiac death, death resulting from heart failure, and death from cardiac procedures. All deaths were deemed cardiac related unless proved otherwise. | MI was defined according to the third universal definition. | Clinically driven TLR was defined as reintervention of the target lesion due to presence of a symptomatic ≥50% diameter stenosis during follow-up.  Angiographically driven TLR was defined as reintervention due to angiographic detection of significant restenosis (≥70%) in a patient who was clinically asymptomatic.  TVR was defined as any revascularization of any segment of the index coronary artery. | 12 months | NA | 12 months | 4 years |
| NBBS IV  FU 2 years  2020 | Provisional vs. Culotte | Cardiac death, non-procedural MI, TLR or definite ST | Cardiac death was defined as death from coronary artery disease including myocardial infarction, sudden death with a possible or definite cardia cause, death from heart failure including cardiogenic shock, and death related to a cardiac procedure within 28 days from the procedure. Cardiac death did not include death due to pulmonary embolism, cerebrovascular attacks or other vascular but non-cardiac events. | Non-procedural myocardial infarction required evidence of myocardial necrosis by at least one of the following criteria: (1) detection of a rise and/or fall of cardiac biomarkers with at least one value above the 99th percentile of the URL and evidence of ischemia in the myocardium documented by either symptoms of ischemia, ECG changes indicative of acute ischaemia (new ST-T changes, new left bundle branch block (LBBB), new pathological Q waves in the ECG), evidence of new loss of viable myocardium or new cardiac wall motion abnormality. (2) Sudden and unexpected cardiac death with at least one of the following: cardiac arrest, symptoms suggestive of myocardial ischemia, presumably new ST-segment elevation, or new LBBB, and/or evidence of fresh thrombus by coronary angiography and/or at autopsy. (3) Pathological findings suggestive of acute myocardial infarction. Assessment of procedural cardiac biomarkers was recommended.  In patients with normal baseline biomarker values, elevations of cardiac biomarkers (CK-MB) greater than 3×99th percentile URL defined index procedure-related myocardial infarction. Patients with stable angina pectoris or silent ischemia were considered to have normal baseline markers if values were not assessed. If cardiac biomarkers were elevated before the procedure and not stable in two samples 6 hours apart, the diagnosis of periprocedural myocardial biomarker increase could not be made. If biomarker values were stable or falling, a 20% or more increase of the value in the second sample after the procedure was required. Elevations of CK-MB greater than 3×99th percentile URL and greater than 5×99th percentile URL were assessed independently. | Target lesion revascularization was defined as repeat revascularization by PCI or coronary artery bypass surgery of the target lesion defined as the stented or balloon-treated segments and their 5 mm margins in all three coronary branches. | 12 months | NA | 8 months | 2 years |

**MI, myocardial infarction; TVR, target vessel revascularization; TLR, target lesion revascularization; TBR, target bifurcation revascularization; TVF, target vessel failure; TLF, target lesion failure; ST, stent thrombosis; CABG, coronary artery bypass graft; DAPT, dual antiplatelet therapy; URL, upper reference limit; ECG, electrocardiogram; STEMI, ST-segment elevation myocardial infarction; NSTEMI, non-ST-segment elevation myocardial infarction; PCI, percutaneous coronary intervention; MV, main vessel; MB, main branch; SB, side branch; IVUS, intravenous ultrasound.**

**Supplemental Table S5: Lesion characteristics and quantitative coronary angiography at baseline**

| Study | Only  true bifurca-tion | Medina  classification | Technique | PMV RVD  (mm) | PMV MLD  (mm) | PMV  DS% | PMV  lesion length  (mm) | DMV RVD  (mm) | DMV MLD  (mm) | DMV  DS% | DMV  lesion length  (mm) |
| --- | --- | --- | --- | --- | --- | --- | --- | --- | --- | --- | --- |
| Colombo et al.  2004 | Yes | NA | T/TAP vs. Provisional | 2.6 ± 0.4  2.6 ± 0.5 | 0.99 ± 0.35  0.92 ± 0.31 | 61.7 ± 12.5  64.7 ± 11.2 | 10.8 ± 4.8  12.2 ± 5.6 | NA | NA | NA | NA |
| Pan et al.  2004 | Yes | 1,1,1/0,1,1 | Provisional vs. T/TAP | 3 ± 0.4  2.9 ± 0.3 | 0.74 ± 0.50  0.76 ± 0.38 | 77 ± 14  74 ± 11 | NA | NA | NA | NA | NA |
| DKCRUSH-I  2008 | NA | NA | Crush vs.  DK Crush | 2.86 ± 0.63  2.85 ± 0.53 | 0.94 ± 0.57  0.93 ± 0.49 | 64.5 ± 13.9  64.8 ± 13.2 | 20.0 ± 9.7  21.3 ± 11.3 | 2.56 ± 0.49  2.53 ± 0.41 | 0.62 ± 0.25  0.62 ± 0.25 | 62.1 ±11.7  65.6 ± 19.7 | NA |
| CACTUS  2009 | Yes | 1,1,1/1,0,1/  0,1,1 | Crush vs. Provisional | 2.85 ± 0.33  2.74 ± 0.35 | 0.90 ± 0.38  0.83 ± 0.33 | 68 ± 12  69 ± 12 | 15.8 ± 8.7  14.7 ± 8.2 | NA | NA | NA | NA |
| THUEBIS  2009 | Yes | 1,1,1/1,0,0/  0,1,0/1,1,0 | Dedicated vs. Provisional | 2.7 ± 0.5  2.7 ± 0.5 | 0.9 ± 0.3  1.0 ± 0.3 | 65.2 ± 8.2  63.5 ± 7.9 | 6.1 ± 2.8  5.1 ± 2.1 | NA | NA | NA | NA |
| BBC ONE  2010 | NA | NA | Provisional vs.  Crush | NA | NA | 87 ± 10  85 ± 11 | NA | NA | NA | NA | NA |
| Lin et al.  2010 | Yes | NA | Provisional vs. DK Crush | 3.98 ± 0.43  3.94 ± 0.44 | 1.65 ± 0.40  1.60 ± 0.35 | 60.00± 8.29  59.92 ± 7.67 | 23.76 ± 2.58  23.56 ± 2.13 | 3.91 ± 0.55  3.82 ± 0.52 | 1.45 ± 0.25  1.43 ± 0.23 | 62.93 ± 4.10  62.47 ± 4.48 | NA |
| Ye et al.  2010 | Yes | 1,1,1/1,0,1/  0,1,1 | DK Crush vs. Provisional | 3.1 ± 0.6  3.3 ± 0.7 | 2.0 ± 0.7  2.1 ± 0.7 | NA | 12.6 ± 7.2  12.3 ± 5.1 | 2.4 ± 0.5  2.8 ± 0.7 | 1.4 ± 0.6  1.6 ± 0.6 | NA | 29.3 ± 8.3  25.3 ± 7.7 |
| Ye et al.  2012 | Yes | 1,1,1/1,0,1/  0,1,1 | DK Crush vs. Provisional | 2.89 ± 0.49  2.83 ± 0.48 | 0.97 ± 0.33  0.95 ± 0.35 | 64.5 ± 7.50  64.8 ± 8.70 | 28.9 ± 11.1  25.5 ± 10.1 | NA | NA | NA | NA |
| NSTS  2013 | Yes | 1,1,1/1,0,1/  0,1,1 | Crush vs. Culotte | 3.38 ± 0.38  3.32 ± 0.33 | NA | NA | 17.4 ± 10.3  17.4 ± 10.1 | NA | NA | NA | NA |
| NBS  2013 | NA | NA | Provisional vs.  Crush | 2.93 ± 0.66  3.00 ± 0.70 | 1.43 ± 0.78  1.62 ± 0.87 | 40 ± 27  46 ± 27 | 18.0 ± 8.3  17.5 ± 7.5 | 2.41± 0.59  2.63± 0.59 | 1.18 ± 0.65  1.32 ± 0.74 | 52 ± 24  50 ± 25 | NA |
| Ruiz et al.  2013 | Yes | NA | Provisional vs. T/TAP | 2.94 ± 0.54  2.91 ± 0.44 | 1.15 ± 0.77  1.10 ± 0.65 | 62.4 ± 22.7  61.8 ± 22.5 | NA | 2.34 ± 0.66  2.42 ± 0.63 | 1.26 ± 0.72  1.35 ± 0.62 | 47.3 ± 21.3  45.4 ± 24.5 | NA |
| DKCRUSH-III  2015 | Yes | 1,1,1/0,0,1 | DK Crush vs. Culotte | NA | 1.47 ± 0.43  1.49 ± 0.42 | 59.84 ± 9.35  59.18 ± 8.47 | 6.36 ± 3.69  6.97 ± 3.86 | NA | 1.09 ± 0.42  1.07 ± 0.44 | 64.64 ± 5.66  65.69 ± 6.46 | 16.67 ± 9.23  18.65 ± 12.26 |
| BBK I  2015 | Yes | NA | Provisional vs. T/TAP | 3.08 ± 0.40  3.08 ± 0.38 | 1.53 ± 0.86  1.63 ± 0.86 | 50.3 ± 26.7  47.3 ± 26.1 | 21.7 ± 7.5  20.9 ± 8.2 | NA | 1.28 ± 0.71  1.20 ± 0.67 | 53.4 ± 24.4  54.9 ± 24.3 | NA |
| TRYTON  2015 | Yes | 1,1,1/1,0,1/  0,1,1 | Dedicated vs. Provisional | 2.91 ± 0.36  2.91 ± 0.35 | 0.99 ± 0.37  1.01 ± 0.35 | 66.16 ± 11.87  65.46 ± 11.10 | 16.8 ± 7.3  16.0 ± 6.8 | NA | NA | NA | NA |
| PERFECT  2015 | Yes | NA | Crush vs. Provisional | 3.6 ± 0.4  3.7 ± 0.5 | 1.1 ± 0.4  1.1 ± 0.4 | 64.4 ± 12.3  65.9 ± 11.7 | 28.9 ± 14.6  27.8 ± 13.1 | 2.6 ± 0.4  2.6 ± 0.4 | NA | NA | NA |
| BBK II  2016 | NA | NA | Culotte vs.  T/TAP | 3.13 ± 0.49  3.15 ± 0.46 | 1.28 ± 0.76  1.29 ± 0.78 | 58.8 ± 23.4  58.9 ± 23.7 | 23.9 ± 7.6  22.7 ± 7.3 | 2.93 ± 0.51  2.96 ± 0.48 | 1.24 ± 0.78  1.19 ± 0.71 | 58.8 ± 23.4  58.9 ± 23.7 | NA |
| EBC TWO  2016 | Yes | NA | Provisional vs. Culotte | NA | 1.10 ± 0.50  1.10 ± 0.49 | 51.3 ± 21.1  47.7 ± 20.5 | 18.0 ± 6.7  18.0 ± 8.8 | NA | NA | NA | NA |
| SMART  2016 | NA | NA | Provisional vs. T/TAP | 3.01 ± 0.45  3.07 ± 0.58 | 0.77 ± 0.48  0.82 ± 0.47 | 74.6 ± 15.2  73.4 ± 14.6 | 13.2 ± 6.9  13.7 ± 8.1 | NA | NA | NA | NA |
| Zhang et al.  2016 | Yes | 1,1,1/1,0,1/  0,1,1 | Provisional vs. Culotte | 3.38 ± 0.56  3.29 ± 0.49 | 1.29 ± 0.88  1.16 ± 0.81 | 61.42 ± 26.99  64.93 ± 24.54 | 7.64 ± 4.86  8.13 ± 4.54 | 3.06 ± 0.49  3.01 ± 0.42 | 0.76 ± 0.62  0.85 ± 0.62 | 75.21 ± 20.62  71.69 ± 19.87 | 14.73 ± 7.70  15.78 ± 8.59 |
| Zheng et al.  2016 | Yes | 1,1,1/1,0,1/  0,1,1 | Crush vs. Culotte | 3.4 ± 0.4  3.3 ± 0.5 | 1.85 ± 0.49  1.87 ± 0.46 | 56.06 ± 8.72  57.19 ± 10.13 | 16.1 ± 6.3  18.5 ± 7.6 | NA | 1.42 ± 0.45  1.47 ± 0.38 | 63.93 ± 7.85  66.33 ± 9.37 | NA |
| DKCRUSH-II  2017 | Yes | 1,1,1/0,1,1 | DK Crush vs. Provisional | 2.86 ± 0.31  2.82 ± 0.37 | 0.94 ± 0.35  0.86 ± 0.36 | 67.2 ± 14.5  69.5 ± 16.9 | 28.4 ± 12.9  28.7 ± 15.5 | NA | NA | NA | NA |
| COBRA  2018 | Yes | 1,1,1/1,0,1/  0,1,1 | Dedicated vs. Culotte | 3.38 ± 0.51  3.39 ± 0.47 | 1.86 ± 0.69  1.46 ± 0.68 | 57 ± 13  62 ± 14 | 7.35 ± 3.66  10.11 ± 3.67 | 2.56 ± 0.48  2.49 ± 0.61 | 1.32 ± 0.48  1.51 ± 0.63 | 48 ± 16  39 ± 17 | 10.58 ± 7.94  7.63 ± 5.75 |
| DKCRUSH-V  2019 | Yes | 1,1,1/0,1,1 | Provisional vs. DK Crush | 3.08 ± 0.45  3.12 ± 0.51 | 1.17 ± 0.51  1.22 ± 0.55 | 61.8 ± 8.1  60.8 ± 7.2 | 6.9 ± 3.5  7.0 ± 3.4 | NA | NA | NA | 6.7 ± 12.5  15.5 ± 12.8 |
| POLBOS I  2020 | No | NA | Dedicated vs.  Provisional | 3.74 ± 0.24  3.61 ± 0.31 | NA | 56 ± 16  62 ± 17 | 8.5 ± 2.4  8.9 ± 2.7 | 3.10 ± 0.38  3.17 ± 0.27 | NA | 69 ± 13  64 ± 11 | 5.8 ± 1.9  7.5 ± 3.4 |
| POLBOS II  2020 | No | NA | Dedicated vs.  Provisional | 3.59 ± 0.23  3.48 ± 0.29 | 1.40 ± 0.24  1.43 ± 0.27 | 61 ± 14  59 ± 16 | 9.3 ± 3.4  9.7 ± 3.7 | 3.01 ± 0.12  3.07 ± 0.23 | 1.56 ± 0.18  1.57 ± 0.19 | 48 ± 16  49 ± 16 | 8.3 ± 2.9  9.8 ± 3.4 |
| NBBS IV  2020 | Yes | 1,1,1/1,0,1/  0,1,1 | Provisional vs. Culotte | 3.13 ± 0.47  3.20 ± 0.57 | 1.29 ± 0.55  1.41 ± 0.60 | 59 ± 16  57 ± 17 | 20.8 ± 9.9  19.5 ± 8.5 | 2.57 ±0.46  2.61 ± 0.54 | 1.43 ± 0.55  1.43 ± 0.58 | 40 ± 20  43 ± 22 | NA |

| Study | SB RVD  (mm) | SB MLD  (mm) | SB DS% | SB  lesion length  (mm) |
| --- | --- | --- | --- | --- |
| Colombo et al.  2004 | 2.1 ± 0.3  2.1 ± 0.3 | 0.88 ± 0.39  1.14 ± 0.52 | 56.8 ± 17.5  46.2 ± 22.3 | 5.5 ± 4.1  5.1 ± 4.4 |
| Pan et al.  2004 | 2.5 ± 0.3  2.5 ± 0.2 | 0.93 ± 0.44  0.85 ± 0.43 | 64 ± 13  65 ± 14 | NA |
| DKCRUSH-I  2008 | 2.45 ± 0.45  2.46 ± 0.54 | 0.84 ± 0.51  0.84 ± 0.58 | 65.7 ± 18.9  65.4 ± 19.8 | 10.5 ± 7.5  10.3 ± 6.3 |
| CACTUS  2009 | 2.30 ± 0.31  2.16 ± 0.33 | 0.84 ± 0.32  0.83 ± 0.30 | 63 ± 12  61 ± 13 | 5.9 ± 4.7  5.7 ± 4.2 |
| THUEBIS  2009 | 2.0 ± 0.6  2.1 ± 0.5 | 1.2 ± 0.5  1.4 ± 0.4 | 41.9 ± 19.9  31.9 ± 19.1 | 2.5 ± 1.2  2.2 ± 1.2 |
| BBC ONE  2010 | NA | NA | 63 ± 31  68 ± 21 | NA |
| Lin et al.  2010 | 2.82 ± 0.25  2.79 ± 0.17 | 0.85 ± 0.16  0.84 ± 0.14 | 69.72 ± 5.28  70.13 ± 4.63 | 12.91 ± 3.12  12.69 ± 2.75 |
| Ye et al.  2010 | 2.2 ± 0.4  2.4 ± 0.7 | 1.4 ± 0.6  1.6 ± 0.6 | NA | 17.1 ± 8.0  11.5 ± 6.9 |
| Ye et al.  2012 | 2.27 ± 0.34  2.39 ± 0.50 | 0.97 ± 0.34  1.01 ± 0.30 | 69.9 ± 7.20  61.0 ± 8.30 | 16.87 ± 8.17  10.24 ± 8.40 |
| NSTS  2013 | 2.78 ± 0.33  2.77 ± 0.33 | NA | NA | 7.3 ± 5.8  7.5 ± 6.0 |
| NBS  2013 | 2.24 ± 0.46  2.28 ± 0.51 | 1.21 ± 0.61  1.22 ± 0.62 | 46 ± 26  47 ± 26 | 6.0 ± 4.8  6.4 ± 4.7 |
| Ruiz et al.  2013 | 2.08 ± 0.56  2.08 ± 0.56 | 1.10 ± 0.44  1.23 ± 0.61 | 45.1 ± 20.5  42.3 ± 23.9 | NA |
| DKCRUSH-III  2015 | NA | 1.01 ± 0.43  1.07 ± 0.49 | 65.29 ± 7.34  63.36 ± 7.75 | 16.48 ± 11.09  16.97 ± 13.01 |
| BBK I  2015 | 2.39 ± 0.31  2.38 ± 0.37 | 1.13 ± 0.62  1.11 ± 0.64 | 53.1 ± 23.5  54.4 ± 22.3 | 10.4 ± 4.1  9.9 ± 4.2 |
| TRYTON  2015 | 2.25 ± 0.30  2.21 ± 0.33 | 0.95 ± 0.34  1.02 ± 0.34 | 58.00 ± 14.28  54.01 ± 14.46 | 4.8 ± 1.6  4.4 ± 1.1 |
| PERFECT  2015 | 2.2 ± 0.4  2.2 ± 0.4 | 1.1 ± 0.4  1.2 ± 0.4 | 57.2 ± 14.5  53.3 ± 16.5 | 10.3 ± 8.2  8.3 ± 7.3 |
| BBK II  2016 | 2.68 ± 0.45  2.62 ± 0.47 | 0.85 ± 0.54  0.82 ± 0.55 | 68.5 ± 18.6  69.5 ± 18.8 | 13.8 ± 6.6  15.5 ± 6.9 |
| EBC TWO  2016 | NA | 0.96 ± 0.37  0.93 ± 0.31 | 54.1 ± 15.6  54.8 ± 13.9 | 9.7 ± 7.1  10.8 ± 7.3 |
| SMART  2016 | 2.46 ± 0.55  2.49 ± 0.54 | 1.29 ± 0.69  1.27 ± 0.71 | 48.8 ± 21.9  50.0 ± 22.9 | 4.4 ± 4.5  4.9 ± 4.0 |
| Zhang et al.  2016 | 2.44 ± 0.40  2.56 ± 0.30 | 0.59 ± 0.30  0.56 ± 0.33 | 76.22 ± 11.60  77.79 ± 12.61 | 12.80 ± 4.92  14.10 ± 7.12 |
| Zheng et al.  2016 | 2.6 ± 0.3  2.7 ± 0.4 | 1.23 ± 0.34  1.32 ± 0.29 | 55.62 ± 10.42  56.25 ± 11.81 | 7.9 ± 4.1  7.4 ± 4.3 |
| DKCRUSH-II  2017 | 2.38 ± 0.32  2.29 ± 0.35 | 0.89 ± 0.30  0.84 ± 0.30 | 62.8 ± 14.7  63.4 ± 14.2 | 15.4 ± 11.3  14.9 ± 12.5 |
| COBRA  2018 | 2.33 ± 0.33  2.21 ± 0.35 | 0.94 ± 0.40  1.19 ± 0.60 | 59 ± 18  46 ± 23 | 9.17 ± 5.91  7.13 ± 5.31 |
| DKCRUSH-V  2019 | 2.69 ± 0.44  2.68 ± 0.41 | 1.02 ± 0.43  1.03 ± 0.45 | 65.3 ± 8.3  65.8± 7.5 | 16.6 ± 11.9  16.2 ± 14.0 |
| POLBOS I  2020 | 2.65 ± 0.29  2.43 ± 0.41 | NA | 61 ± 17  54 ± 21 | 3.7 ± 1.3  2.9 ± 1.8 |
| POLBOS II  2020 | 2.45 ± 0.39  2.34 ± 0.34 | 1.13 ± 0.20  1.29 ± 0.16 | 54 ± 24  45 ± 18 | 4.2 ± 2.1  3.9 ± 2.8 |
| NBBS IV  2020 | 2.33 ± 0.49  2.40 ± 0.49 | 1.43 ± 0.69  1.21 ± 0.46 | 43 ± 18  49 ± 17 | 2.9 ± 0.2  2.9 ± 0.2 |

**PMV: proximal main vessel; RVD: reference vessel diameter; MLD: minimal luminal diameter; DS: diameter stenosis; DMV: distal main vessel; SB: side branch; T/TAP: T-stenting/T-stenting and protrusion; DK: double-kissing.**

**Supplemental Table S6: Lesion characteristics and quantitative coronary angiography after procedure**

| Study | Technique | PMV RVD  (mm) | PMV MLD  (mm) | PMV  DS% | DMV RVD  (mm) | DMV MLD  (mm) | DMV  DS% | SB RVD  (mm) | SB MLD  (mm) | SB DS% |
| --- | --- | --- | --- | --- | --- | --- | --- | --- | --- | --- |
| Colombo et al.  2004 | T/TAP vs. Provisional | 3.0 ± 0.4  3.0 ± 0.4 | 2.66 ± 0.40  2.65 ± 0.35 | 11.5 ± 7.7  11.7 ± 7.7 | NA | NA | NA | 2.5 ± 0.3  2.2 ± 0.4 | 2.11 ± 0.44  1.69 ± 0.63 | 14.4 ± 13.8  23.5 ± 27.2 |
| Pan et al.  2004 | Provisional vs. T/TAP | NA | 2.75 ± 0.38  2.66 ± 0.33 | 10 ± 8  9 ± 9 | NA | NA | NA | NA | 1.95 ± 0.52  2.15 ± 0.45 | 21 ± 17  12 ± 14 |
| DKCRUSH-I  2008 | Crush vs.  DK Crush | 3.44 ± 0.54  3.48 ± 0.51 | 2.94 ± 0.55  3.02 ± 0.52 | 12.8 ± 7.1  14.6 ± 8.0 | 3.15 ± 0.57  3.26 ± 0.55 | 2.67 ± 0.56  2.96 ± 0.57 | 14.3 ± 6.4  13.5 ± 5.9 | 2.85 ± 0.39  2.88 ± 0.40 | 2.56 ± 0.42  2.59 ± 0.45 | 19.4 ± 8.7  14.6 ± 8.7 |
| CACTUS  2009 | Crush vs. Provisional | 2.99 ± 0.47  2.87 ± 0.42 | 2.71 ± 0.32  2.58 ± 0.33 | 12 ± 6  13 ± 6 | NA | NA | NA | 2.43 ± 0.36  2.24 ± 0.35 | 1.94 ± 0.39  1.65 ± 0.39 | 16 ± 11  27 ± 14 |
| THUEBIS  2009 | Dedicated vs. Provisional | NA | 2.3 ± 0.4  2.3 ± 0.4 | 21.7 ± 8.8  21.7 ± 8.3 | NA | NA | NA | NA | 1.5 ± 0.5  1.3 ± 0.5 | 31.3 ± 18.0  38.8 ± 19.9 |
| BBC ONE  2010 | Provisional vs.  Crush | NA | NA | NA | NA | NA | NA | NA | NA | NA |
| Lin et al.  2010 | Provisional vs. DK Crush | 4.01 ± 0.41  3.99 ± 0.42 | 3.57 ± 0.55  3.51 ± 0.41 | 14.25 ± 3.86  13.14 ± 5.81 | 4.02 ± 0.50  3.95 ± 0.53 | 3.43 ± 0.54  3.35 ± 0.51 | 14.83 ± 4.95  15.12 ± 5.23 | 2.89 ± 0.27  2.87 ± 0.16 | 2.37 ± 0.40  2.42 ± 0.18 | 17.92 ± 10.81  15.38 ± 6.18 |
| Ye et al.  2010 | DK Crush vs. Provisional | NA | 2.8 ± 0.3  2.9 ± 0.5 | 6.3 ± 6.4  7.8 ± 7.1 | NA | 2.5 ± 0.3  2.7 ± 0.5 | 8.0 ± 5.7  7.9 ± 6.8 | NA | 2.2 ± 0.3  2.0 ± 0.6 | 9.1 ± 6.2  15.0 ± 12.2 |
| Ye et al.  2012 | DK Crush vs. Provisional | NA | NA | NA | NA | NA | NA | NA | NA | NA |
| NSTS  2013 | Crush vs. Culotte | 3.51 ± 0.55  3.44 ± 0.52 | 3.18 ± 0.54  3.14 ± 0.55 | 9.16 ± 8.22  8.54 ± 10.21 | 2.87 ± 0.50  2.87 ± 0.47 | 2.57 ± 0.48  2.56 ± 0.46 | 10.43 ± 10.59  10.36 ± 9.42 | 2.64 ± 0.58  2.61 ± 0.49 | 2.25 ± 0.49  2.28 ± 0.44 | 13.79 ± 12.78  12.22 ± 10.93 |
| NBS  2013 | Provisional vs.  Crush | 3.21 ± 0.52  3.30 ± 0.51 | 2.86 ± 0.55  3.04 ± 0.51 | 11 ± 10  7± 10 | 2.69 ± 0.45  2.83 ± 0.46 | 2.34 ± 0.44  2.50 ± 0.46 | 13 ± 13  11 ± 10 | 2.28 ± 0.44  2.47 ± 0.46 | 1.50 ± 0.64  2.05 ± 0.54 | 34 ± 23  16 ± 18 |
| Ruiz et al.  2013 | Provisional vs. T/TAP | NA | 3.00 ± 0.63  2.94 ± 0.51 | 6.2 ± 6.3  7.0 ± 6.0 | NA | 2.61 ± 0.54  2.46 ± 0.47 | 11.5 ± 8.4  10.8 ± 6.7 | NA | 1.66 ± 0.45  2.03 ± 0.44 | 20.8 ± 14.3  14.1 ± 9.2 |
| DKCRUSH-III  2015 | DK Crush vs. Culotte | NA | NA | 11.08 ± 7.24  11.81 ± 6.83 | NA | NA | 16.15 ± 8.33  15.41 ± 7.10 | NA | NA | 16.39 ± 7.45  25.50 ± 7.36 |
| BBK I  2015 | Provisional vs. T/TAP | NA | 3.22 ± 0.45  3.17 ± 0.51 | 2.52 ± 9.34  3.03 ± 10.7 | NA | 2.77 ± 0.39  2.74 ± 0.41 | 7.64 ± 8.63  9.32 ± 9.73 | NA | 1.97 ± 0.46  2.30 ± 0.43 | 16.6 ± 13.9  9.56 ± 11.4 |
| TRYTON  2015 | Dedicated vs. Provisional | 2.99 ± 0.37  2.97 ± 0.36 | 2.71 ± 0.38  2.70 ± 0.34 | 9.33 ± 7.43  8.97 ± 7.54 | NA | NA | NA | 2.31 ± 0.33  2.25 ± 0.33 | 2.36 ± 0.32  NA | -2.21 ± 10.12  NA |
| PERFECT  2015 | Crush vs. Provisional | NA | 2.6 ± 0.4  2.7 ± 0.4 | 13.5± 7.2  13.0 ± 6.9 | NA | NA | NA | NA | 2.3 ± 0.4  1.9 ± 0.6 | 13.7 ± 11.1  25.7 ± 17.8 |
| BBK II  2016 | Culotte vs.  T/TAP | 3.33 ± 0.45  3.37 ± 0.44 | 3.12 ± 0.43  3.16 ± 0.46 | 6.30 ± 5.01  6.52 ± 5.49 | 3.20 ± 0.44  3.20 ± 0.42 | 2.97 ± 0.44  2.98 ± 0.41 | 7.31 ± 5.68  7.11 ± 5.63 | 2.91 ± 0.45  2.81 ± 0.38 | 2.67 ± 0.44  2.60 ± 0.40 | 8.17 ± 6.39  7.32 ± 6.39 |
| EBC TWO  2016 | Provisional vs. Culotte | NA | 2.46 ± 0.46  2.33 ± 0.38 | 10.8 ± 7.6  8.0 ± 8.3 | NA | NA | NA | NA | 1.53 ± 0.43  2.03 ± 0.35 | 31.2 ± 13.8  25.1 ± 11.1 |
| SMART  2016 | Provisional vs. T/TAP | 3.17 ± 0.45  3.21 ± 0.53 | 2.28 ± 0.54  2.27 ± 0.51 | 28.2 ± 12.2  29.4 ± 10.5 | NA | NA | NA | 2.39 ± 0.50  2.48 ± 0.54 | 1.34 ± 0.58  1.57 ± 0.57 | 45.0 ± 15.9  37.0 ± 15.3 |
| Zhang et al.  2016 | Provisional vs. Culotte | 3.48 ± 0.62  3.33 ± 0.50 | 3.24 ± 0.59  3.13 ± 0.48 | 7.02 ± 5.66  6.11 ± 4.86 | 3.19 ± 0.53  3.04 ± 0.42 | 2.88 ± 0.53  2.74 ± 0.43 | 9.97 ± 5.71  9.87 ± 6.01 | 2.45 ± 0.40  2.74 ± 0.35 | 2.01 ± 0.61  2.40 ± 0.35 | 18.73 ± 16.52  12.30 ± 7.43 |
| Zheng et al.  2016 | Crush vs. Culotte | NA | NA | 8.95 ± 5.31  8.43 ± 4.92 | NA | NA | 11.48 ± 7.93  11.26 ± 6.69 | NA | NA | 14.26 ± 11.20  13.02 ± 10.45 |
| DKCRUSH-II  2017 | DK Crush vs. Provisional | 2.97 ± 0.44  2.89 ± 0.41 | 2.72 ± 0.51  2.58 ± 0.44 | 9.7 ± 3.7  11.9 ± 6.3 | NA | NA | NA | 2.49 ± 0.38  2.36 ± 0.35 | 2.18 ± 0.43  1.63 ± 0.46 | 12.3 ± 8.60  28.6 ± 13.8 |
| COBRA  2018 | Dedicated vs. Culotte | 3.50 ± 0.46  3.29 ± 0.49 | 3.21 ± 0.52  2.95 ± 0.34 | NA | 2.60 ± 0.40  2.36 ± 0.40 | 2.32 ± 0.45  2.37 ± 0.43 | NA | 2.25 ± 0.35  2.16 ± 0.35 | 2.03 ± 0.36  2.12 ± 0.23 | NA |
| DKCRUSH-V  2019 | Provisional vs. DK Crush | 3.09 ± 0.44  3.14 ± 0.51 | 2.65 ± 0.46  2.73 ± 0.49 | 14.3 ± 8.2  13.0 ± 7.9 | NA | NA | NA | 2.74 ± 0.45  2.76 ± 0.48 | 2.01 ± 0.50  2.49 ± 0.48 | 25.2 ± 15.0  8.70 ± 2.10 |
| POLBOS I  2020 | Dedicated vs.  Provisional | 3.78 ± 0.23  3.68 ± 0.22 | NA | 5± 7  7± 12 | 3.15 ± 0.28  3.24 ± 0.12 | NA | 8± 11  4± 9 | 2.69 ± 0.24  2.43 ± 0.32 | NA | 45 ± 21  39 ± 14 |
| POLBOS II  2020 | Dedicated vs.  Provisional | 3.68 ± 0.19  3.53 ± 0.25 | 3.28 ± 0.15  3.25 ± 0.19 | 11 ± 4  8± 9 | 3.10 ± 0.38  3.15 ± 0.32 | 2.67 ± 0.27  2.74 ± 0.21 | 14 ± 12  13 ± 8 | 2.41 ± 0.14  2.39 ± 0.22 | 1.64 ± 0.20  1.72 ± 0.15 | 32 ± 19  28 ± 21 |
| NBBSIV  2020 | Provisional vs. Culotte | NA | 3.05 ± 1.46  2.84 ± 1.48 | 11 ± 9  8± 7 | NA | 2.45 ± 0.42  2.51 ± 0.41 | 11 ± 9  11 ± 9 | NA | 1.59 ± 0.65  2.10 ± 0.37 | 36 ± 19  17 ± 11 |

**PMV: proximal main vessel; RVD: reference vessel diameter; MLD: minimal luminal diameter; DS: diameter stenosis; DMV: distal main vessel; SB: side branch; T/TAP: T-stenting/****T-stenting and protrusion; DK: double-kissing.**

**Supplemental Table S7: Sensitivity analysis including only true bifurcation**

**Comparison for MACEs**

| **Provisional** | 1.12  (0.74-1.75) | 0.95  (0.64-1.47) | **0.37**  **(0.24-0.55)** | 1.25  (0.70-2.09) | 1.19  (0.64-2.11) |
| --- | --- | --- | --- | --- | --- |
| 0.89  (0.57-1.36) | **Crush** | 0.83  (0.53-1.35) | **0.34**  **(0.18-0.57)** | 1.11  (0.54-2.13) | 1.07  (0.49-2.14) |
| 1.06  (0.68-1.56) | 1.20  (0.74-1.88) | **Culotte** | **0.40**  **(0.22-0.63)** | 1.32  (0.65-2.49) | 1.26  (0.58-2.52) |
| **2.69**  **(1.81-4.17)** | **2.94**  **(1.76-5.51)** | **2.51**  **(1.59-4.44)** | **DK Crush** | **3.36**  **(1.69-6.54)** | **3.21**  **(1.54-6.52)** |
| 0.80  (0.48-1.43) | 0.90  (0.47-1.86) | 0.76  (0.40-1.55) | **0.30**  **(0.15-0.59)** | **Dedicated** | 0.94  (0.42-2.29) |
| 0.84  (0.47-1.56) | 0.93  (0.47-2.05) | 0.79  (0.40-1.71) | **0.31**  **(0.15-0.65)** | 1.06  (0.44-2.36) | **T-stenting/TAP** |

**MACE: major adverse cardiovascular event; DK: double-kissing; TAP: T-stenting and protrusion.**

**Results are the ORs in the column-defining treatment compared with the ORs in the row-defining treatment. For outcomes, ORs higher than 1 favor the row-defining treatment. Significant results are in bold and underscored.**

**Supplemental Table S8: Sensitivity analysis excluding studies at high-risk bias and only including studies at low-risk bias**

| **Provisional** | 1.28  (0.92-1.76) | 0.97  (0.65-1.46) | **0.40**  **(0.26-0.58)** | 1.42  (0.70-2.62) | 1.50  (0.89-2.51) |
| --- | --- | --- | --- | --- | --- |
| 0.66  (0.27-1.57) | **Crush** | 0.75  (0.50-1.16) | **0.31**  **(0.20-0.48)** | 1.10  (0.51-2.21) | 1.17  (0.64-2.10) |
| 0.86  (0.21-3.42) | 1.31  (0.44-3.93) | **Culotte** | **0.41**  **(0.25-0.65)** | 1.47  (0.67-2.99) | 1.55  (0.86-2.73) |
| 1.71  (0.70-4.26) | **2.60**  **(1.06-6.61)** | 2.00  (0.49-8.15) | **DK Crush** | **3.54**  **(1.64-7.39)** | **3.76**  **(2.02-7.10)** |
| 0.86  (0.05-14.21) | 1.32  (0.10-18.31) | 1.02  (0.09-11.52) | 0.50  (0.03-8.59) | **Dedicated** | 1.06  (0.48-2.53) |
| 0.48  (0.08-3.21) | 0.74  (0.15-3.89) | 0.56  (0.17-1.88) | 0.28  (0.04-1.87) | 0.55  (0.04-8.12) | **T-stenting/TAP** |

**Comparison for MACEs**

**MACE: major adverse cardiovascular event; DK: double-kissing; TAP: T-stenting and protrusion.**

**Results are the ORs in the column-defining treatment compared with the ORs in the row-defining treatment. For outcomes, ORs higher than 1 favor the row-defining treatment. Significant results are in bold and underscored.**

**Supplemental Figure S1: Subgroups rank probability analysis for MACEs**

**
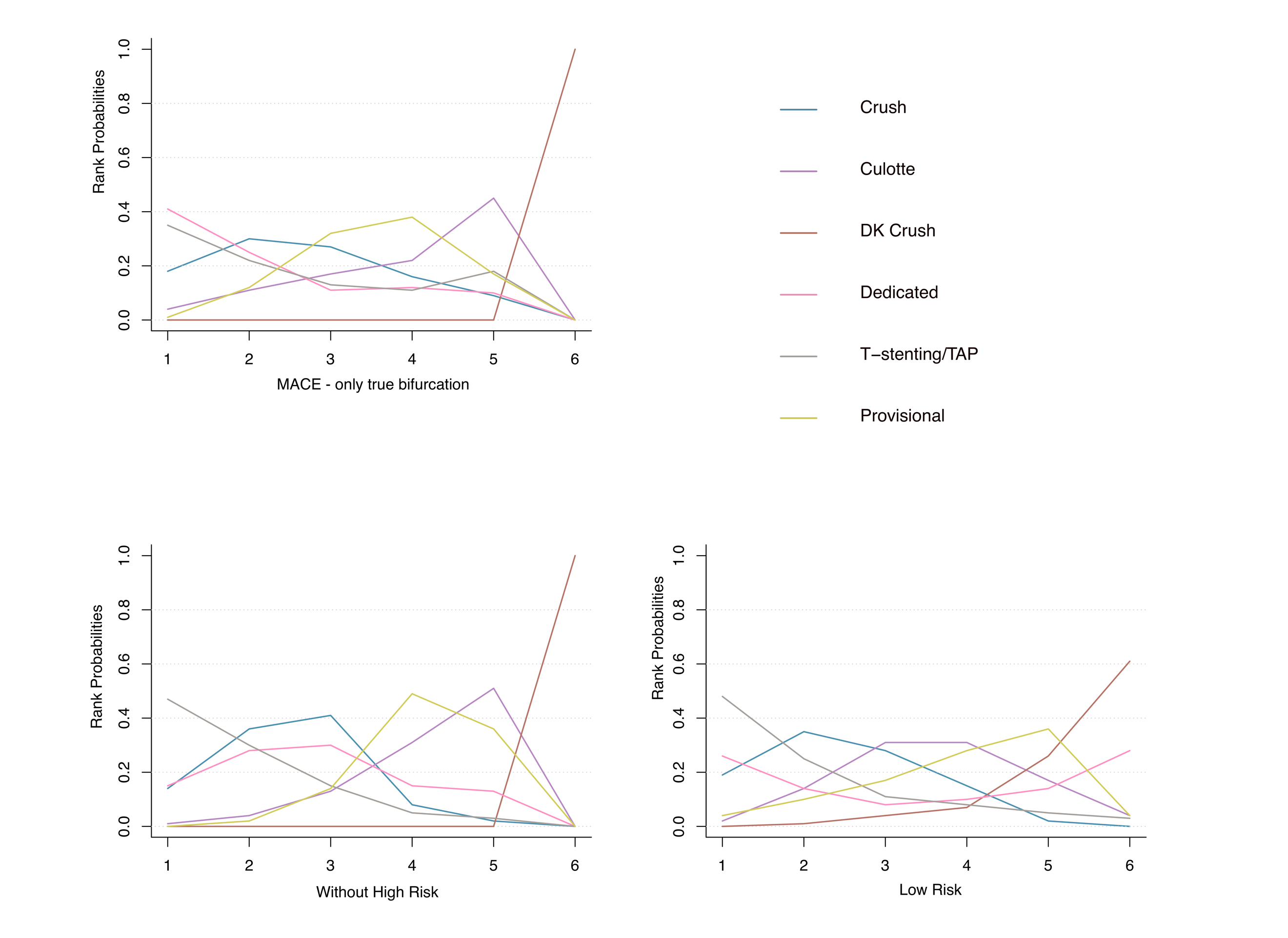
**

**MACE: major adverse cardiovascular event; DK: double-kissing; TAP: T-stenting and protrusion.**

**Rank 1 is identified as the treatment with the highest incidence of each endpoint events and Rank 6 is identified as the treatment with the lowest incidence of each endpoint events.**
